# Mice Deficient in the Respiratory Chain Gene *Cox6a2* Are Protected against High-Fat Diet-Induced Obesity and Insulin Resistance

**DOI:** 10.1371/journal.pone.0056719

**Published:** 2013-02-27

**Authors:** Roel Quintens, Sarvjeet Singh, Katleen Lemaire, Katrien De Bock, Mikaela Granvik, Anica Schraenen, Irene Olga Cornelia Maria Vroegrijk, Veronica Costa, Pieter Van Noten, Dennis Lambrechts, Stefan Lehnert, Leentje Van Lommel, Lieven Thorrez, Geoffroy De Faudeur, Johannes Anthonius Romijn, John Michael Shelton, Luca Scorrano, Henri Roger Lijnen, Peter Jacobus Voshol, Peter Carmeliet, Pradeep Puthenveetil Abraham Mammen, Frans Schuit

**Affiliations:** 1 Gene Expression Unit, Department of Molecular and Cellular Medicine, Katholieke Universiteit Leuven, Leuven, Belgium; 2 Division of Cardiology, Department of Internal Medicine, University of Texas Southwestern Medical Center, Dallas, Texas, United States of America; 3 Vesalius Research Center, Katholieke Universiteit Leuven, Leuven, Belgium; 4 Vesalius Research Center, Vlaams Instituut voor Biotechnologie (VIB), Leuven, Belgium; 5 Department of Endocrinology and Metabolic Diseases, Leiden University Medical Center, Leiden, The Netherlands; 6 Department of Cell Physiology and Metabolism, University of Geneva, Geneve, Switzerland; 7 Physical Activity and Health Laboratory, Biomedical Kinesiology Department, Katholieke Universiteit Leuven, Leuven, Belgium; 8 Department of Metallurgy and Materials Engineering, KU Leuven, Leuven, Belgium; 9 Center for Molecular and Vascular Biology, Katholieke Universiteit Leuven, Leuven, Belgium; Mayo Clinic, United States of America

## Abstract

Oxidative phosphorylation in mitochondria is responsible for 90% of ATP synthesis in most cells. This essential housekeeping function is mediated by nuclear and mitochondrial genes encoding subunits of complex I to V of the respiratory chain. Although complex IV is the best studied of these complexes, the exact function of the striated muscle-specific subunit COX6A2 is still poorly understood. In this study, we show that *Cox6a2*-deficient mice are protected against high-fat diet-induced obesity, insulin resistance and glucose intolerance. This phenotype results from elevated energy expenditure and a skeletal muscle fiber type switch towards more oxidative fibers. At the molecular level we observe increased formation of reactive oxygen species, constitutive activation of AMP-activated protein kinase, and enhanced expression of uncoupling proteins. Our data indicate that COX6A2 is a regulator of respiratory uncoupling in muscle and we demonstrate that a novel and direct link exists between muscle respiratory chain activity and diet-induced obesity/insulin resistance.

## Introduction

The worldwide prevalence of type 2 diabetes and obesity has reached epidemic proportions as the result of the interaction of a Westernized lifestyle with genetic determinants that are prevalent in human populations. Obesity often predisposes to the development of insulin resistance and type 2 diabetes, and therefore weight loss by promotion of a healthy lifestyle is often the initial therapeutic target of intervention for obese, type 2 diabetic patients. However, this strategy has only short-term success in 95% of the cases [1]. Thus, it has been proposed that long-term strategies for inducing weight loss should also aim at increasing the patients' metabolic rates (energy expenditure) by decreasing their metabolic efficiency. This can be achieved in mice by induction of mitochondrial uncoupling proteins (UCPs), which dissipate energy as heat [2]–[5]. UCPs can be induced by different mechanisms among which reactive oxygen species (ROS) [6], or via activation of AMP-activated protein kinase (AMPK) [7], [8], which is a master regulator of energy homeostasis.

The role of mitochondrial dysfunction in the etiology of insulin resistance, type 2 diabetes and obesity is still unclear. Early reports indicated that mitochondrial dysfunction was a causative factor [9]–[12] and was the result of the accumulation of metabolites such as ROS and long chain fatty acids [13]–[15]. However, over the past few years, several studies have suggested that changes in mitochondrial function may be compensatory or even protective, rather than a cause of these diseases [16]–[18]. Moreover, it seems that mild mitochondrial dysfunction in muscle and liver in mice protects against obesity and diabetes [19], whereas mice with a progressive muscle-specific respiratory chain deficiency have reduced blood glucose levels and increased peripheral glucose uptake [20].

COX6A protein is one of the thirteen subunits of the respiratory chain complex IV (cytochrome *c* oxidase). Its exact role in this complex is not yet known; possible functions may be assembly of the complex as well as regulation of the catalytic properties of the core subunits. Mammalian COX6A is represented by two different isoforms, COX6A-L (COX6A1, liver-type) and COX6A-H (COX6A2, heart-type), which are encoded in the mouse by the *Cox6a1* and *Cox6a2* genes, respectively. Two differences between these isoforms are their expression and regulation. The COX6A1 subunit is ubiquitously expressed, whereas expression of COX6A2 is restricted to striated muscles [21], [22]. Unlike COX6A1, the COX6A2 subunit has the capability to bind ADP and ATP [23]. Binding of ADP results in an increase in catalytic activity which can be abolished by competition with a monoclonal antibody [24]. Moreover, at high intramitochondrial ATP/ADP ratios (i.e. at rest), reconstituted bovine heart complex IV has decreased proton pumping capacity, also referred to as intrinsic uncoupling or “slip” [25]. This mechanism has been suggested to play a role in thermogenesis in heart and skeletal muscle at rest [26] as well as in the protection against the formation of mitochondrial ROS [27], [28]. Interestingly, in the stepwise assembly of the mammalian complex IV, the COX6A subunit is only added in the final step [29], suggesting that this subunit plays a major role in the expression and enzymatic activity of functional complex IV.

Until present, the functional consequence of a global deletion of the *Cox6a2* gene in mice (*Cox6a2* ^−/−^) has only been investigated for the heart: the phenotype was described as mild diastolic cardiac dysfunction at elevated workload, resulting in decreased stroke work [30]. Because *Cox6a2* is also expressed in skeletal muscle and because of the specific regulatory properties of COX6A2 subunit in oxidative phosphorylation, we were interested in the effect of COX6A2 deficiency on skeletal muscle function as well as whole body energy metabolism and glucose homeostasis. Our data demonstrate a previously unrecognized link between presence of the *Cox6a2* gene in mice and the susceptibility to develop high-fat diet-induced obesity, insulin resistance and glucose intolerance.

## Results

### COX6A2 deficiency results in a moderate decrease in complex IV enzymatic activity and increased production of ROS in skeletal muscles

**Figure 1:**
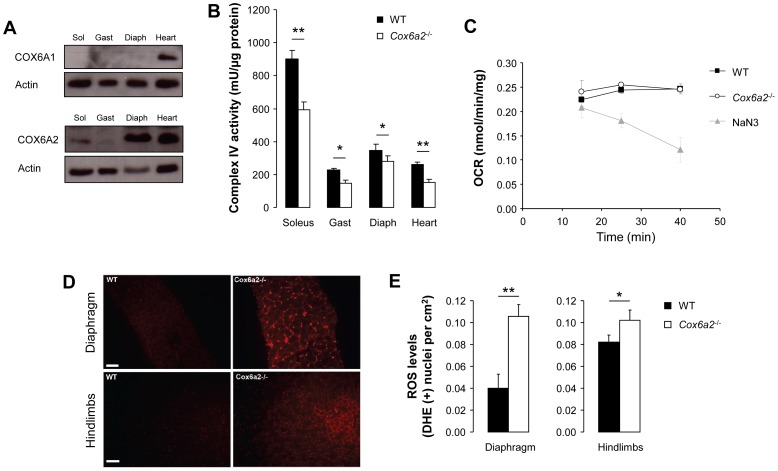
COX6A1 and COX6A2 expression in skeletal muscles and heart of WT mice. Disruption of the gene leads to reduced complex IV activity and enhanced ROS generation. (A) Representative images of western blots for COX6A1 and COX6A2 in different skeletal muscles. COX6A1 expression was only observed in the heart, whereas COX6A2 protein expression is highest in the heart and diaphragm and lowest in the gastrocnemius muscle, suggesting that COX6A2 expression correlates with muscle oxidative capacity. Sol: soleus muscle; Gast: gastrocnemius muscle; Diaph: diaphragm, (B) Complex IV activity measurements in skeletal muscles and heart of WT and *Cox6a2* ^−/−^ mice (n = 3), (C) Oxygen consumption rate in diaphragm of WT vs *Cox6a2* ^−/−^ mice (n = 3). Inhibition of complex IV by NaN_3_ was used to validate the method, (D) Measurement of steady state ROS levels by DHE staining. Representative images of DHE stained sections of the diaphragm (upper) and hindlimbs (lower) of WT and *Cox6a2* ^−/−^ mice. (E) Quantification of the number of positive myocytes stained with DHE (n = 6). *p<0.05, **p<0.01. Data represent mean+SEM.

In agreement with previous reports [31], [32], COX6A1 protein expression was very low in three types of skeletal muscle: soleus (slow oxidative, mainly type I fibers), gastrocnemius (fast glycolytic, mainly type IIB fibers) and diaphragm (fast oxidative, mainly type IIA fibers) (Fig. 1A). On the contrary, COX6A2 protein was abundant not only in heart, but also in diaphragm and to a lesser extent in soleus and gastrocnemius muscle (Fig. 1A). In order to assess a direct functional consequence of *Cox6a2* deletion in mice, we measured mitochondrial complex IV enzymatic activity, both in heart and skeletal muscle and observed a moderate but significant reduction in all of these tissues (Fig. 1B). In contrast, oxygen consumption rates of isolated diaphragm were not affected by the *Cox6a2* deletion (Fig. 1C), indicating the existence of compensatory mechanisms. Since reduced complex IV activity is often associated with enhanced ROS production, and because intrinsic uncoupling of oxidative phosphorylation has been proposed to protect against the formation of ROS [28] we also measured steady state ROS levels in the diaphragm and hindlimbs of *Cox6a2* ^−/−^ and WT mice utilizing dihydroethidium, which preferentially indicates superoxide [33]. As is demonstrated in figures 1D and E, elevated ROS levels were detected both in diaphragm and in hindlimb muscles of *Cox6a2* ^−/−^ mice as compared to WT mice with the largest difference being measured in the diaphragm. Together, our results indicate that COX6A2 deficiency causes moderate loss of complex IV activity and increased ROS production in skeletal muscle.

### *Cox6a2* ^−/−^ mice are lean and protected against diet-induced obesity

**Figure 2:**
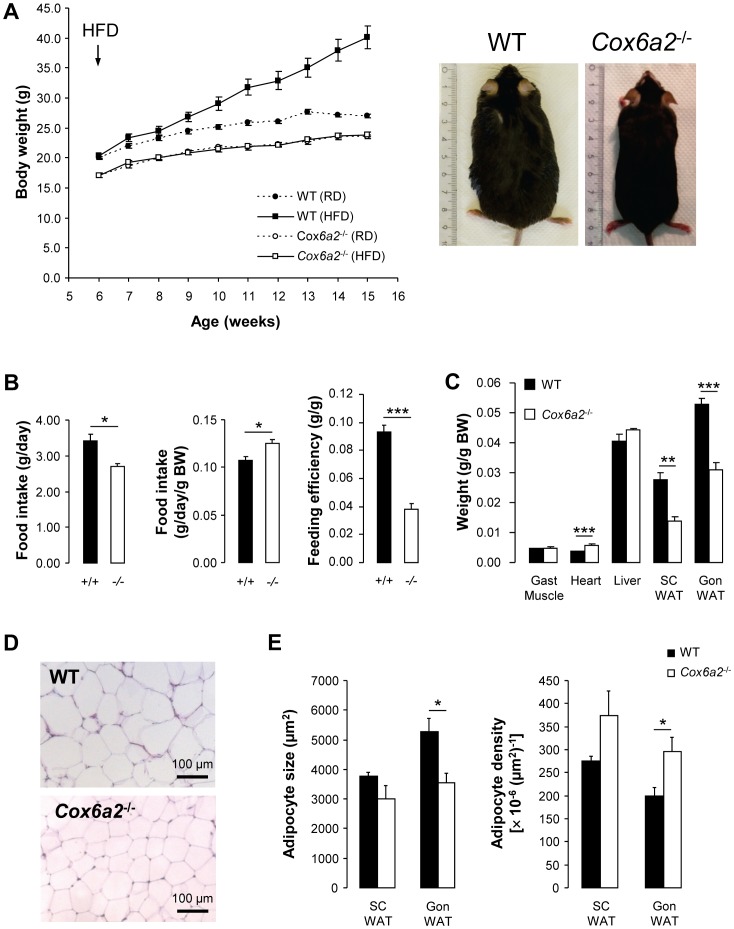
*Cox6a2* ^−/−^ mice are protected against high-fat diet-induced obesity. (A) Mice were fed a regular diet or a HFD starting from 6 weeks of age (n = 5 for WT, n = 14–16 for *Cox6a2* ^−/−^ mice). Body weight was monitored weekly. The right panel shows representative images of WT and *Cox6a2* ^−/−^ mice after 12 weeks of HFD feeding, (B) Absolute food intake, relative food intake and feed efficiency of mice fed a HFD (n = 5), (C) Average weight of gastrocnemius muscle, heart, liver, subcutaneous WAT and gonadal WAT dissected from mice that were fed a HFD for 12 weeks (n = 5). (D) Representative pictures of H&E staining of gonadal WAT of mice fed a HFD for 12 weeks. (E) Quantification of subcutaneous and gonadal WAT cell size and density (n = 5). *p<0.05, **p<0.01, ***p<0.001. In all panels, data represent mean+SEM.

Accumulation of ROS in skeletal muscle is often associated with mitochondrial dysfunction, insulin resistance and obesity. Therefore, we investigated the metabolic phenotype of *Cox6a2* ^−/−^ mice and compared animals that were fed a regular diet to those fed a high-fat diet (HFD). As shown in figure 2A, WT mice gained extra weight when fed a HFD as compared to a regular diet. However, *Cox6a2* ^−/−^ mice had superimposable weight gain curves on regular diet or HFD (Fig. 2A). Absolute food intake was reduced in HFD-fed *Cox6a2* ^−/−^ mice (Fig. 2B, left panel) but when corrected for body weight, food intake was significantly increased in *Cox6a2* ^−/−^ mice (Fig. 2B, middle panel). The difference in weight gain between WT and *Cox6a2* ^−/−^ mice can be attributed to decreased feed efficiency in the *Cox6a2* ^−/−^ strain (Fig. 2B, right panel). The increment in whole body weight after a HFD was primarily caused by increased mass of adipose tissue (both in gonadal and subcutaneous fat pads) in control animals (Fig. 2C). We also observed slightly increased heart weights in *Cox6a2* ^−/−^ mice (Fig. 2C) in which conforms the previous observation of cardiac hypertrophy caused by diastolic dysfunction [30].

The reduced mass of the gonadal fat pad in *Cox6a2* ^−/−^ mice fed a HFD was associated with decreased adipocyte size and increased cellular density 12 weeks after the start of HFD feeding (Fig. 2D, E). A similar trend was observed in adipocytes from subcutaneous fat (Fig. 2E). Therefore, at the whole organism level, deficiency of COX6A2 protects against HFD-induced fat mass accumulation.

### Increased glucose tolerance and insulin sensitivity in *Cox6a2* ^−/−^ mice fed a HFD

**Figure 3:**
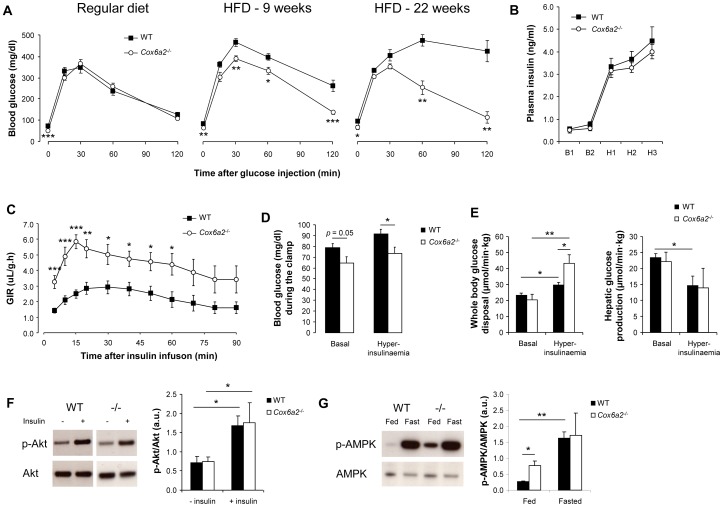
Increased glucose tolerance and insulin sensitivity in *Cox6a2* ^−/−^ mice is associated with constitutive activation of AMPK. (A) After a 16–18 h fast, mice were injected with 2.5 mg/g BW glucose and blood glucose levels were monitored for 2 h (n = 7–9 for RD, n = 4–11 for 9 weeks of HFD, n = 3–4 for 22 weeks of HFD). Note that WT animals become progressively glucose intolerant when receiving HFD, whereas *Cox6a2* ^−/−^ mice are completely protected against HFD-induced glucose intolerance, (B) Hyperinsulinemic, euglycemic clamps were performed on mice (21 weeks old) fed a HFD for 15 weeks (n = 6–8). Plasma insulin levels before (10 min: B_1_; 0 min: B_2_) and after (70 min: H_1_; 80 min: H_2_; 90 min: H_3_) insulin infusion, (C) The glucose infusion rate (GIR) was monitored for 90 min after administration of a hyperinsulinemic solution via the tail vein, (D) Blood glucose levels before insulin infusion (basal) and at the end of the clamp (hyperinsulinaemia), (E) Whole body glucose disposal (left) and hepatic glucose production (right) were measured during the basal period and under hyperinsulinemic conditions, (F) Western blot analysis of insulin-stimulated phosphorylation of Akt in soleus muscle of regular diet fed mice. No difference was observed between fasted (18 h) WT and *Cox6a2* ^−/−^ mice (n = 3), (G) Western blot analysis of AMPK phosphorylation in response to fasting. Mice (n = 3) on a regular diet were either fed *ad libitum* or fasted overnight (18 h) before dissection of soleus muscles. *p<0.05, **p<0.01, ***p<0.001. In all panels, data represent mean ± SEM.

Development of diet-induced obesity in WT mice was associated with increased plasma levels of insulin, leptin, cholesterol and triglycerides as compared to *Cox6a2* ^−/−^ mice (Table S1). Despite similar random fed blood glucose levels, the difference in plasma insulin levels between WT and *Cox6a2* ^−/−^ mice suggested that a difference in insulin sensitivity existed between the two strains. To further investigate this idea, we first performed intraperitoneal glucose tolerance tests in mice that were either on a regular diet or a HFD for 9 or 22 weeks. Although *Cox6a2* ^−/−^ mice fed a regular diet had significantly lower fasting glucose levels, no difference in glucose tolerance between *Cox6a2* ^−/−^ and WT mice was observed (Fig. 3A, left panel). However, while WT mice fed a HFD developed progressive glucose intolerance with a marked degree of hyperglycemia two hours after glucose injection (Fig. 3A), no difference in glucose tolerance was observed in *Cox6a2* ^−/−^ mice after 22 weeks of HFD *versus* regular diet (Fig. 3A). To assess whether the preservation of glucose tolerance in *Cox6a2* ^−/−^ mice after HFD was a result of increased insulin sensitivity, we performed hyperinsulinemic, euglycemic clamps on mice fed a HFD for 15 weeks. During hyperinsulinemia, plasma insulin levels were comparable between WT and *Cox6a2* ^−/−^ mice (Fig. 3B). Within 5 min after start of the i.v. administration of insulin, *Cox6a2* ^−/−^ mice exhibited a significant drop in blood glucose levels, required a significantly higher glucose infusion rate (Fig. 3C) to remain euglycemic but nevertheless had significantly lower blood glucose during the clamp than those in control mice (Fig. 3D). During hyperinsulinemia, whole body glucose disposal increased by 25% in WT mice as compared to the basal state (Fig. 3E). In *Cox6a2* ^−/−^ mice, however, whole body glucose disposal was more than 2-fold increased (Fig. 3E) during the clamp, indicating that peripheral (80% skeletal muscle) insulin sensitivity was significantly enhanced in *Cox6a2* ^−/−^ mice as compared to WT mice. Hepatic glucose production (HGP) was comparable in WT and *Cox6a2* ^−/−^ mice, which suggested that hepatic insulin sensitivity was not affected by the loss of the gene (Fig. 3E). We also performed clamps on mice fed a regular diet (WT: 26.2±1.6 g BW; *Cox6a2* ^−/−^: 22.8±0.4 g BW; *p* = 0.056). Unfortunately, four out of five *Cox6a2* ^−/−^ mice died during the experiment because of excessive glucose infusion rates that could not prevent hypoglycemia. The average glucose level of these mice at the time of death was 42.3±4.7 mg/dl. This suggested that *Cox6a2* ^−/−^ mice are more insulin sensitive as compared to WT mice, even when fed a regular diet. As a possible mechanism, we examined the activities of the insulin signaling intermediate Akt and of the metabolic regulator AMPK. Whereas we found no difference in insulin-stimulated phosphorylation of Akt (Fig. 3F), we observed that phosphorylation of AMPK was increased about threefold in skeletal muscle of regular fed *Cox6a2* ^−/−^ mice (Fig. 3G) indicating that AMPK is constitutively activated in *Cox6a2* ^−/−^ mice.

### Energy expenditure and adaptive thermogenesis are increased in *Cox6a2* ^−/−^ mice

**Figure 4:**
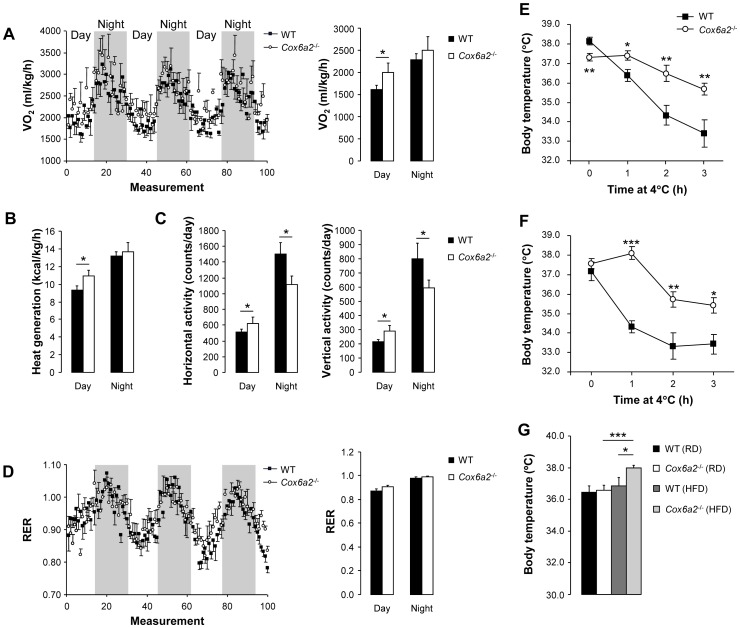
Increased energy expenditure and adaptive thermogenesis in *Cox6a2* ^−/−^ mice. (A–D) Oxygen consumption (A, left panel), average oxygen consumption (A, right panel), heat generation (B), spontaneous activity (C), and respiratory exchange ratio (D, left panel), average respiratory exchange ratio (D, right panel), measured in weight-matched mice of 12–16 weeks old (n = 4–5). Measurements were made over a three day period. Gray shades in A and B indicate dark cycles, (E–F) Mice (12–14 weeks old) fed a regular diet (E) or a HFD (F) were deprived of food, exposed to cold (4°C) and core body temperature was measured every hour for three hours (n = 5–9 for regular diet, n = 5–11 for HFD), (G) Core body temperature of mice (12–14 weeks old) fed a regular or a HFD (n = 5–9 for regular diet, n = 5–11 for HFD). *p<0.05, **p<0.01, ***p<0.001. In all panels, data represent mean ± SEM.

In order to determine whether the lean phenotype of the *Cox6a2* ^−/−^ mice was due to an increased basal metabolic rate and energy expenditure, indirect calorimetric measurements were performed on weight-matched male mice fed a regular diet. The body composition of these mice was assessed using MRI, and revealed a small but statistically significant decrease in percent lean body mass in the *Cox6a2* ^−/−^ mice as compared to WT mice (Fig. S1A), whereas there was no difference in % fat mass in mice fed a regular diet (Fig. S1B). Daily food intake was similar between *Cox6a2* ^−/−^ and WT mice, but the daily intake of water was slightly lower in *Cox6a2* ^−/−^ mice (Fig. S1C). The indirect calorimetric measurements showed that, compared to WT mice, *Cox6a2* ^−/−^ mice consumed more oxygen and generated more heat during the day (Fig. 4A, B), which confirms an increased metabolic rate in *Cox6a2* ^−/−^ mice compared to WT mice. During the night, there was a non-significant increase in oxygen consumption and heat generation in *Cox6a2* ^−/−^ mice, despite a 25% decrease in spontaneous activity compared to WT mice (Fig. 4C). The calorimetric studies also revealed no difference in the respiratory exchange ratio (RER) between WT and *Cox6a2* ^−/−^ mice (Fig. 4D), suggesting that the ratio of carbohydrate over fat oxidation is unaltered in *Cox6a2* ^−/−^ mice. These data also indicated that the increased energy expenditure and thermogenesis in *Cox6a2* ^−/−^ mice were the main reasons for the lean phenotype of these mice, rather than differences in fuel selection for mitochondrial oxidation.

We also assessed the effect of COX6A2 deficiency on adaptive thermogenesis by exposing mice to cold (4°C) for three hours and monitoring body temperature. Body temperature remained relatively constant in *Cox6a2* ^−/−^ mice fed either a regular diet (Fig. 4E) or a HFD (Fig. 4F), indicating enhanced non-shivering thermogenesis. Moreover, we also found that, in contrast to WT mice, *Cox6a2* ^−/−^ mice fed a HFD had increased core body temperature as compared to mice fed a regular diet (Fig. 4G). This may represent an additional mechanism to explain the resistance to diet-induced obesity we observed in *Cox6a2* ^−/−^ mice.

To rule out a compensatory thermoregulatory mechanism via the pituitary-thyroid axis, we also assessed thyrotropin (TSH) bioactivity (a measure for TSH content) and serum T_4_ levels. These assays revealed that both hormones were not significantly altered in *Cox6a2* ^−/−^ mice (Fig. S1D), suggesting that the pituitary-thyroid axis was not responsible for the increased thermogenesis observed in *Cox6a2* ^−/−^ mice exposed to cold. Therefore we wondered whether the COX6A2 subunit might be also expressed in thermogenic brown adipose tissue (BAT) or subcutaneous white adipose tissue (SC WAT), which has recently been shown to contain brown-like cells, called “beige fat” [34]. Thus, we analyzed public microarray data of BAT (GSE7623, [35]) and SC WAT (E-MEXP-1636, [36]). In both data sets, we found very heterogeneous Cox6a2 signals (Fig. S2) which may be reflective of either sample contamination, or expression of the gene in only a subpopulation of the cells, as would be expected in SC WAT. In the case of the latter, a high degree of correlation in the expression between Cox6a2 and the thermogenic protein UCP1 was to be expected. However, whereas expression of UCP1 was also very heterogeneous among the different SC WAT samples, there was no correlation with the expression of Cox6a2 (Fig. S2B). Instead, both in BAT and SC WAT we found very strong correlation between Cox6a2 and skeletal muscle markers (Fig. S2), indicating that when Cox6a2 expression is found in these tissues, it is most likely the result of contaminating skeletal muscle tissue in the samples.

Together, these data indicate that increased thermogenesis in *Cox6a2* ^−/−^ mice, which may be partly responsible for their protection against diet-induced obesity, results from loss of the gene in skeletal muscles, rather than more established thermogenic tissues such as BAT. These results also indicate that the COX6A2 subunit is an important regulator for whole-body metabolic rate.

### *Cox6a2* ^−/−^ mice lose body weight faster upon short-term starvation

The protection from diet-induced obesity, the inefficient energy metabolism and the increase in energy expenditure predicted that *Cox6a2* ^−/−^ mice would tolerate starvation less well than control mice. We tested this prediction by a starvation experiment with free access to water for 36 h at room temperature. The observation was that both male and female *Cox6a2* ^−/−^ mice lost significantly more weight compared to WT animals (Fig. S3A, B). In fact, two out of ten *Cox6a2* ^−/−^ male mice had to be refed after 24 h, because they were lethargic. Between 24 h and 36 h of fasting, the calculated average weight loss in the remaining eight *Cox6a2* ^−/−^ male mice (slope = −0.52±0.03% weight loss per h) was 2-fold greater as compared to the male controls (slope = −0.28±0.02%; *p*<0.001) (Fig. S3A). This period corresponds to the “coping phase” of body weight loss, in which primarily fat and ketone bodies are used as an energy source [37]. We also evaluated the effect of fasting on *Cox6a2* expression in different muscles and found that during fasting, *Cox6a2* expression, both at the mRNA (Fig. S3C) as well as the protein level (Fig. S3D), was increased in the soleus muscle but not the gastrocnemius or the diaphragm of WT mice. This suggests that the feeding-dependent regulation of *Cox6a2* expression is restricted to type I myofibers.

### *Cox6a2* ^−/−^ mice display elevated expression of uncoupling proteins in muscles, heart and adipose tissue

**Figure 5:**
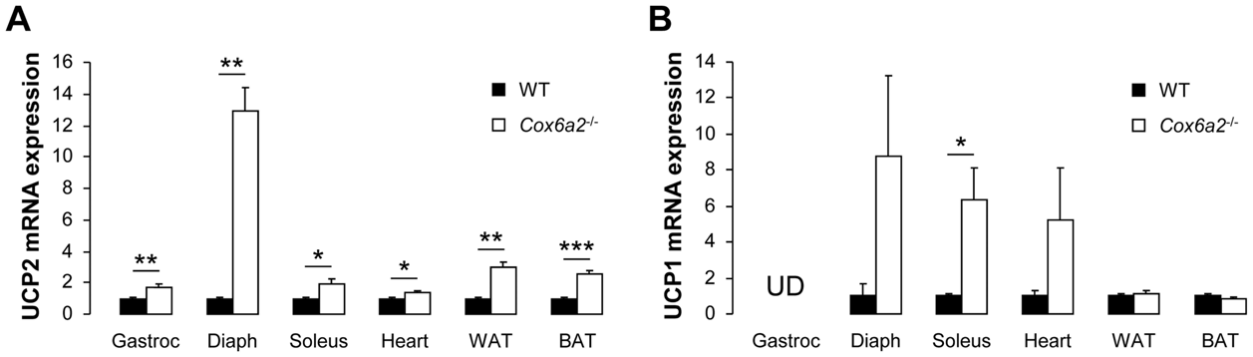
Increased UCP2 (A) and UCP1 (B) expression in metabolically active tissues of *Cox6a2* ^−/−^ mice. Quantitative RT-PCRs were performed on cDNA from gastrocnemius muscle, diaphragm, soleus muscle, heart, white and brown adipose tissue (n = 3–5). Gene expression in WT mice was set at 1.0 for each individual tissue. UD = Undetectable. Please note that UCP1 mRNA expression in BAT is about 100-fold that of other tissues. *p<0.05, **p<0.01, ***p<0.001. In all panels, data represent mean ± SEM.

Uncoupling proteins serve a critical role in the regulation of cell metabolism and overall energy expenditure and are often induced in animal models that are protected against obesity. We therefore measured the *Ucp* transcript levels not only in skeletal muscle, but also in heart, and adipose tissue (WAT and BAT). A significant relative increase in UCP2 expression was measured in muscle and fat of *Cox6a2* ^−/−^ mice, but there were tissue-specific differences (Fig. 5A) with the highest increase being measured in the diaphragm. Furthermore, UCP1 mRNA expression in the diaphragm, soleus muscle and heart of *Cox6a2* ^−/−^ mice was upregulated by 4- to 9-fold (Fig. 5B) In WAT of mice that were fed a HFD we found that both UCP1 and UCP2 mRNA expression was further increased 3-to 5-fold in *Cox6a2* ^−/−^ mice as compared to mice fed a regular diet (Fig. S4A). Interestingly, a similar increase in UCP2 expression in response to HFD was seen in WAT of obesity-resistant C57BL/KsJ and A/J mice, whereas in the obesity-prone C57BL/6J mouse strain no difference in UCP2 expression could be seen in HFD fed mice [38]. Also in gastrocnemius muscle of HFD fed *Cox6a2* ^−/−^ mice, expression of UCP2 was further elevated compared to *Cox6a2* ^−/−^ mice on a regular diet (Fig. S4B).

### Fiber type switch in skeletal muscle of *Cox6a2* ^−/−^ mice

**Figure 6:**
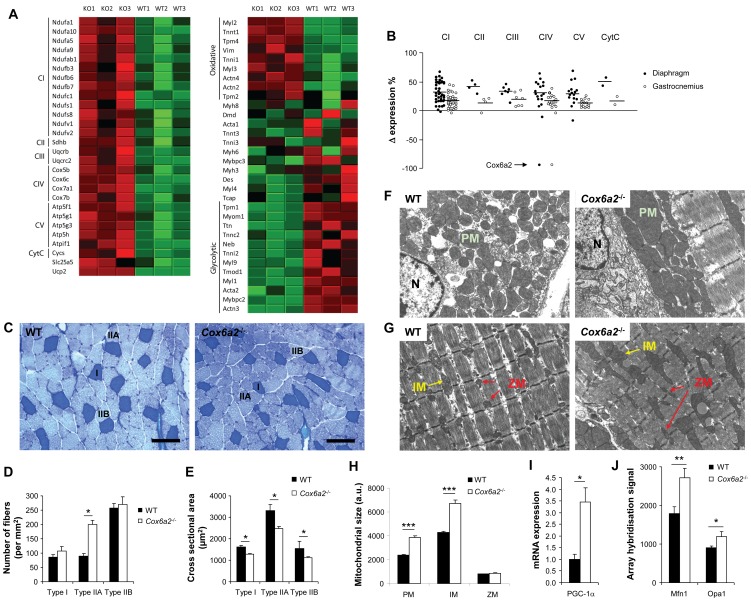
Fiber type switch and increased mitochondrial size in muscles of *Cox6a2* ^−/−^ mice. (A) Heat maps of top subsets of genes of a Gene Set Enrichment Analysis of gene expression in diaphragm of WT *versus Cox6a2* ^−/−^ mice on a regular diet (n = 3). Genes were ranked according to their signal-to-noise ratio. Left: Electron transport chain genes. Right: Striated muscle contraction genes. Red color indicates high expression, green color indicates low expression. KO: *Cox6a2* ^−/−^, (B) Relative changes in expression of genes of the electron transport chain in diaphragm and gastrocnemius muscle of *Cox6a2* ^−/−^ mice compared to WT mice. Horizontal bars indicate mean difference in expression. For complex IV, Cox6a2 was not taken into account for calculation of the mean difference in expression. CI, complex I; CII, complex II; CIII, complex III; CIV, complex IV; CV, complex V (ATP synthase); CytC, cytochrome *c*, (C) Fiber typing of gastrocnemius muscle by a metachromatic ATPase assay. ATPase activity stains type I fibers dark blue, type IIA fibers light blue, and type IIB fibers very light blue. Representative images of fiber typing in the gastrocnemius muscle from WT and *Cox6a2* ^−/−^ mice are shown. Scale bar = 100 µm. I, IIA, and IIB refer to type I, type IIA, and type IIB fibers, (D) Quantification of muscle fiber types in gastrocnemius muscle of WT and *Cox6a2* ^−/−^ mice (n = 20–30). Note the increase in the total number of fibers in gastrocnemius muscle from *Cox6a2* ^−/−^ mice, (E) Cross-sectional area of different fiber types in gastrocnemius of WT and *Cox6a2* ^−/−^ mice (n = 30–60), (F–G) Transmission electron micrographs of WT and *Cox6a2* ^−/−^ diaphragm. Perinuclear (F) and intermyofibrillar mitochondria (G) are shown. N: nucleus; M: mitochondrion; IM: intermyofibrillar mitochondria; Z: small intermyofibrillar mitochondria located near Z-discs, (H) Quantification of the mitochondrial size of the diaphragm of WT and *Cox6a2* ^−/−^ mice, (I) Pgc-1α mRNA expression in diaphragm of WT and *Cox6a2* ^−/−^ mice (n = 3) was measured using qRT-PCR, (J) mRNA expression signals for Mfn1 and Opa1 as measured by microarrays.

The changes in *Ucp* gene expression and ROS levels in muscle of *Cox6a2* ^−/−^ mice led to the question whether more global differences of the gene expression exist. Since most of the changes in mRNA expression were small, a gene set enrichment analysis (GSEA) was undertaken to identify statistically significant, coordinate changes in gene expression of *a priori* defined sets of genes. In both the gastrocnemius muscle and diaphragm, expression of genes associated with mitochondrial function as well as oxidoreductase activity was elevated in *Cox6a2* ^−/−^ mice as compared to WT mice (Table S2). Significant enrichment was found for instance for antioxidant enzymes (Table S3) as well as the subunits of the respiratory chain (Fig. 6A,B) which were, with the obvious exception of *Cox6a2*, almost all slightly (10–70%) upregulated (Fig. 6B). Similarly to the induction of ROS production (Fig. 1E), the effects at the level of gene expression were more outspoken in the diaphragm compared to the gastrocnemius muscle.

The upregulation at the level of the respiratory chain coincided with enhanced expression of markers for oxidative (type I and IIA) myofibers, both in gastrocnemius muscle and in diaphragm of *Cox6a2* ^−/−^ mice (Fig. 6A, right panel). In contrast, markers for glycolytic (type IIB) myofibers were downregulated (Fig. 6A, right panel). These data indicate that in *Cox6a2* ^−/−^ mice, skeletal muscle fibers have switched to a slower, more oxidative phenotype. This change was confirmed at the histochemical level by performing a metachromatic ATPase assay, by which the fiber type composition within a skeletal muscle group can be determined. As is demonstrated in figures 6C and 6D, we observed an increase in the number of oxidative type IIA fibers within the gastrocnemius muscle of *Cox6a2* ^−/−^ mice. Moreover, the total number of myofibers per mm^2^ was significantly increased in *Cox6a2* ^−/−^ gastrocnemius muscle, and the cross-sectional areas of all the fiber types in *Cox6a2* ^−/−^ mice were significantly reduced (Fig. 6E). Interestingly, we found that *Fndc5*, the gene that codes for the recently discovered antidiabetic hormone irisin [39] was also significantly increased in *Cox6a2* ^−/−^ diaphragm (data not shown). This is probably also a result of the fiber type switch since we found in the publicly available data from a microgenomic analysis of individual fast and slow myofibers [40] that *Fndc5* is preferentially expressed in slow fibers.

Together with a change in gene expression and fiber type, we also observed a change in mitochondrial size. Perinuclear as well as intermyofibrillar mitochondria were larger in the diaphragm of *Cox6a2* ^−/−^ mice (Fig. 6F, G, H), although we did not observe a difference in size of the small intermyofibrillar mitochondria located near the Z-discs. The alignment of myofibrils as well as the dense packaging of cristae within the mitochondria were not affected in *Cox6a2* ^−/−^ mice. The increase in mitochondrial size we observed in the diaphragm was associated with increased expression of the peroxisome proliferator-activated receptor γ coactivator, PGC-1α (Fig. 6I), a key regulator of mitochondrial biogenesis [41] and of skeletal muscle fiber type determination [42]. Also mitofusin-1 (Mfn1) and optic atrophy 1 (Opa1), both of which regulate mitochondrial fusion and therefore mitochondrial size [43], were increased in the diaphragm of *Cox6a2* ^−/−^ mice (Fig. 6J).

### Isolated skeletal muscles of *Cox6a2* ^−/−^ mice are more resistance to fatigue

**Figure 7:**
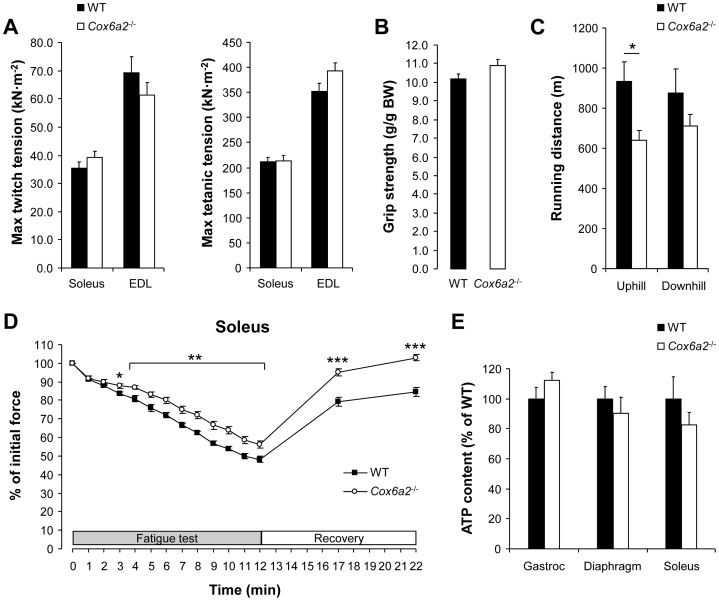
Isolated soleus muscle from *Cox6a2* ^−/−^ mice is more resistant to fatigue. (A) Muscle tension measurements. Twitch (both 1 Hz) and tetanic (50 Hz and 100 Hz) tension were measured from isolated soleus and EDL muscle, respectively (n = 8–10), (B) Grip strength measurements (n = 4–5), (C) 14 week old WT and *Cox6a2* ^−/−^ mice were forced to run up- or downhill until exhaustion. Data are presented as distance (m) ran until exhaustion (n = 5), (D) Muscle force during a fatigue test and after recovery in isolated soleus muscle (n = 8–10), (E) Skeletal muscle ATP content. ATP concentrations were measured in diaphragm, gastrocnemius and soleus muscle of WT and *Cox6a2* ^−/−^ mice (n = 5). *p<0.05, **p<0.01, ***p<0.001. In all panels, data represent mean ± SEM.

The switch in muscle fiber composition towards a more oxidative profile in *Cox6a2* ^−/−^ mice was also reflected by the mechanical properties of these muscles. The specific force production (force/cross-sectional area) of soleus and extensor digitorum longus (EDL) muscles (Fig. 7A), as well as the grip strength (Fig. 7B) were similar between WT and *Cox6a2* ^−/−^ mice. However, in a fatiguing protocol, isolated soleus muscle of *Cox6a2* ^−/−^ mice was more resistant to fatigue and recovered much faster as compared to WT muscle (Fig. 7D), which is consistent with an increase in oxidative muscle fibers. However, this was not reflected at the level of the whole animal since we observed that in an endurance experiment *Cox6a2* ^−/−^ mice had decreased exercise capacity as compared to WT mice when running uphill. When mice were forced to run downhill there was no significant difference in performance, although there was again a tendency towards reduced performance in *Cox6a2* ^−/−^ mice (Fig. 7C). We ascribe this discrepancy between the *in vitro* and *in vivo* muscle performance to the cardiac phenotype of these mice (i.e. diastolic dysfunction), which is only observed under high workloads [30]. Finally, despite the loss of COX6A2 expression, ATP levels within the skeletal muscles of *Cox6a2* ^−/−^ and WT mice were similar (Fig. 7E), which is consistent with previous observations in the heart [30]. Thus, *Cox6a2* ^−/−^ mice have developed compensatory mechanisms that allow production of sufficient amounts of ATP to perform mechanical work.

## Discussion

In this study, we explored the metabolic phenotype of *Cox6a2* ^−/−^ mice and we found a previously unrecognized direct functional link between complex IV of the respiratory chain, and whole body energy metabolism. We propose on basis of these observations that skeletal muscle COX6A2 acts as a key regulator of whole body energy homeostasis, thermogenesis and response to the fasted state. Although COX6A2 protein expression is known to be abundant in heart and skeletal muscle, a previous analysis of *Cox6a2*-deficient mice was restricted to the heart. These mice were reported to be viable but developed mild diastolic dysfunction during high work load [30]. One could speculate that this cardiac phenotype would compromise a metabolic characterization of the mice because it might affect oxygen and nutrient delivery to various tissues. However, *Cox6a2* ^−/−^ mice do not have systolic dysfunction and therefore their circulation is not impaired. As a result, *Cox6a2* ^−/−^ mice do not develop tissue hypoxia due to hypoperfusion, a consequence often seen in both humans and animals with systolic heart failure. As Cox6a2 is both expressed in the heart and in skeletal muscles, our current study focused on the metabolic profile as well as the muscle phenotype of the *Cox6a2* ^−/−^ mice. The present study in mice links *Cox6a2*-deficiency to protection to the metabolic changes of obesity, insulin resistance and glucose intolerance that are observed in control mice after a HFD.

*Cox6a2* ^−/−^ mice are characterized by an elevated metabolic rate which results in increased energy expenditure, thermogenesis and susceptibility to fasting-induced weight loss. We interpret our observations as being caused by loss of COX6A2 expression in skeletal muscle, which is, like BAT, an important tissue for non-shivering thermogenesis [44]. The increase in energy expenditure is observed despite a decrease in spontaneous activity. Although decreased spontaneous activity may be suggestive of additional underlying pathologies, we observed no other obvious behavioral differences between WT and *Cox6a2* ^−/−^ mice. In fact, there are several other mouse models with genetic or pharmacological protection against obesity due to increased energy expenditure which also show decreased physical activity [45]–[50]. Thus, one possible explanation is that mice with a higher basal metabolic rate move less to compensate the energy balance.

A plausible mechanism is that the observed changes in metabolic rate are due to an increase in expression of uncoupling proteins in skeletal muscles and brown as well as white fat. It is already well known from the use of 2,4-dinitrophenol and β_3_-adrenoreceptor agonists that a pharmacological increase of mitochondrial uncoupling leads to weight reduction, but these agents can have very serious side effects. Protective effects against obesity have also been attributed to the uncoupling proteins by means of genetic induction in mice [2]–[5], [51]. UCP1 is mainly expressed in brown fat where it generates heat by uncoupling the mitochondrial proton gradient from ATP production. However, a moderate induction of UCP1 expression in white fat (10% of brown fat levels) [52] or skeletal muscle (1% of brown fat levels) [5] can be sufficient to protect against obesity, even despite a 35% decline in brown fat mass [5]. Recently, it was shown that a subpopulation of subcutaneous white fat cells (beige cells) have endogenous UCP1-dependent thermogenic properties upon stimulation [53]. The exact physiological roles of UCP2 and UCP3, which are more ubiquitously expressed, is still under debate [54]. Both UCP2 and UCP3 knockout mice respond normally to cold exposure and are not obese, at least under room temperature [55]–[57]. However, it has been suggested that UCP2 as well as UCP3 may well be significantly thermogenic when fully activated by endogenous or exogenous effectors such as superoxide [58]. It is also known that ROS can induce the expression [6] as well as the activity of UCPs [59]. Therefore, our observation that the increase in expression of UCPs is associated with elevated skeletal muscle ROS production is significant. Interestingly, expression of UCPs is not only induced in skeletal muscle and the heart of *Cox6a2* ^−/−^ mice, but also in brown and white adipose tissue. This suggests the existence of a crosstalk between skeletal muscle and adipose tissue, as was recently shown with the identification of a new hormone, irisin, which stimulates browning and UCP1 expression in WAT [39]. The *Fndc5* gene, which codes for irisin, seems to be a marker of slow myofibers and is induced in the diaphragm of *Cox6a2* ^−/−^ mice. Whether this increased *Fndc5* expression also results in increased irisin levels in *Cox6a2* ^−/−^ mice remains to be established.

Although the COX6A2 subunit has been proposed to be involved in thermogenesis at rest [26], the *Cox6a2* ^−/−^ mice actually have increased thermogenesis. We therefore hypothesize that the intrinsic uncoupling activity of COX6A2 is compensated for by extrinsic uncoupling via UCPs, which increase thermogenesis in a more constitutive manner. Thus, increased uncoupling via UCPs, is most likely the primary mechanism underlying the protection against obesity in *Cox6a2* ^−/−^ mice. This further corroborates the idea that increasing energy expenditure via uncoupling of ATP synthesis may be an effective method for reducing body weight.

The enhanced ROS levels in muscles of *Cox6a2* ^−/−^ mice, support the hypothesis that proton pump slipping protects against ROS formation in the mitochondria [28]. It was an unexpected observation that skeletal muscles which generate more ROS display increased insulin sensitivity, especially in the setting of data suggesting that elevated ROS levels are associated with mitochondrial dysfunction and insulin resistance [13]–[15]. However, over the past few years, several studies have shown that mild mitochondrial dysfunction both in skeletal muscles, liver and adipose tissues, might even protect against the development of glucose intolerance, insulin resistance and obesity [19], [20], [60] and it has been suggested that mitochondrial dysfunction is a consequence rather than a cause of these conditions [16]–[18]. Also, emerging data supports an important role of ROS as second messengers in the regulation of cell signaling in skeletal muscle. For instance, it has been shown that moderate physical exercise results in increased muscle ROS generation followed by induction of endogenous antioxidant defense mechanisms [61]. This can be prevented by antioxidant intake which negated the health-promoting effects of physical exercise on insulin resistance in humans [62], forming the basis for the concept of mitohormesis [63]. This novel concept suggests that increased ROS production in the mitochondria results in an adaptive antioxidative response which eventually leads to a long-term reduction of oxidative stress. In support of these findings, Tiganis and colleagues revealed that ROS can actually enhance insulin sensitivity and that mice with elevated ROS levels in various tissues can be protected against HFD-induced insulin resistance [64]. Another recent study showed that aging and cellular senescence were accelerated in Tap73 knockout mice. This phenotype resulted from increased ROS production due to a reduction of Cox4i1 expression and subsequent impaired complex IV activity. Importantly, these mice gained less weight and were more insulin sensitive compared to control mice when fed a HFD, although oxygen consumption was decreased [65].

In view of these data, it is interesting to note that it was recently shown that ROS can activate AMPK [66], which is constitutively activated in *Cox6a2* ^−/−^ mice (Fig. 3G). AMPK is a key regulator of energy homeostasis, responding to an elevated [AMP]/[ATP] ratio for instance due to increased mitochondrial uncoupling via UCP1 [8], [67], and initiating a signal cascade that accelerates catabolic pathways while inhibiting anabolic pathways. The AMPK metabolic sensor also activates [7] and induces gene expression [66] of *Pgc-1α*, which can subsequently lead to increased mitochondrial biogenesis as well as induction of PGC-1α target genes such as *Ucp2*, *Ucp3* and *Slc2a4*, which codes for the facilitated glucose transporter GLUT4 [7]. Although we have not been able to identify the exact mechanism of activation of UCPs, AMPK and PGC-1α, we believe that signaling via ROS may play a central role. Indeed, it has been shown that uncoupling proteins [6], AMPK [66] as well as PGC-1α [68] can be directly activated by ROS. Together, our data provide further evidence that increased ROS levels in skeletal muscle are not sufficient to induce insulin resistance and that mild mitochondrial dysfunction may even protect against metabolic disorders.

Another aspect of the phenotype of *Cox6a2* ^−/−^ mice which may be attributed to the increased expression of PGC-1α, was the switch in skeletal muscle fibers towards a more oxidative profile in *Cox6a2* ^−/−^ mice, resulting in more fatigue-resistant skeletal muscles with larger mitochondria. These findings contrast with those observed in mice with a skeletal muscle-specific knockout of *Cox10* which develop a severe, progressive myopathy, with decreased muscle performance resulting in premature death at 3 to 4 months of age [69]. Unlike the *Cox6a2* ^−/−^ mice, however, *Cox10* ^−/−^ mice also displayed a severe reduction in skeletal muscle complex IV activity (below 5% of control) leading to significantly decreased ATP levels (35% of control). The discrepancy between both phenotypes may potentially be explained by cell-free reconstitution experiments: the COX10 subunit is a chaperone needed for the early steps to form the complex; in contrast, a late assembly intermediate forms without COX6A protein, which binds as the last subunit completing the formation of the holoenzyme [29]. These cell-free experiments and the data in the present study suggest that the COX6A subunit plays a role in the regulation of the activity and the expression of the fully functional complex, as the cell would be able to rapidly increase the amount of functional complex IV by synthesizing COX6A alone, rather than all 13 subunits. In agreement with this idea, incomplete complex IV in COX6A knockdown cells retains residual electron transfer potential [29]. Thus, the phenotypic abnormalities in *Cox6a2* ^−/−^ mice are more subtle than a general respiratory chain defect and appear more regulatory in nature.

**Figure 8:**
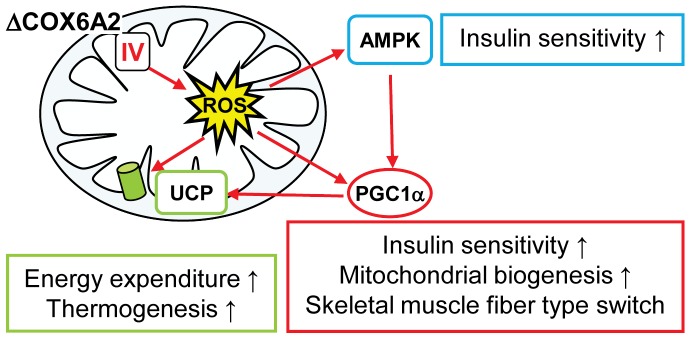
Model explaining the metabolic phenotype of *Cox6a2* ^−/−^ mice. Loss of COX6A2 protein (ΔCOX6A2) in complex IV enhances ROS production by the respiratory chain, which activates AMPK, PGC-1α and upregulates uncoupling protein expression in skeletal muscle. This results in increased energy expenditure, non-shivering thermogenesis, as well as muscle fiber type switch and enhanced insulin sensitivity.

The fiber type switch in *Cox6a2* ^−/−^ mice also partly explains the increased insulin sensitivity and glucose tolerance of the mice. Indeed, oxidative fibers express more of the glucose transporter GLUT4, which is primarily responsible for glucose uptake in skeletal muscles. Moreover, several studies have shown that increased numbers of oxidative fibers (type I and/or type IIA fibers) result in enhanced insulin sensitivity and glucose tolerance [67], [70]–[73]. Another compensatory mechanism, probably related to the fiber type switch, is the coordinate upregulation of virtually all of the nuclear encoded subunits of the respiratory chain in muscles of *Cox6a2* ^−/−^ mice (Fig. 7A and B). This correlates well with the modest increases of complex I, III and V protein that were observed in the heart of *Cox6a2* ^−/−^ mice [30]. A similar induction of genes involved in oxidative phosphorylation, associated with increased mitochondrial mass, was seen in mice treated with the PGC-1α activator resveratrol, which protects mice from developing obesity and insulin resistance [49]. Taken together, the metabolic phenotype that results from loss of COX6A2 protein from complex IV could be modeled as follows (Fig. 8): decreased complex IV activity contributes to increased ROS production which activates AMPK and PGC-1α and which enhances respiratory uncoupling via UCPs. This results in increased thermogenesis and energy expenditure, mitochondrial biogenesis and myofiber type switching, all of which explain why these mice are protected against diet-induced obesity and display enhanced insulin sensitivity. Of course this simplified model does not exclude the role of other potential regulators that are influenced by ROS such as p38, and Keap1/Nrf2. The effect of COX6A2 ablation very much resembles that of calorie restriction/exercise and resveratrol as reviewed by Hoeks and Schrauwen [74]. It is therefore tempting to speculate that (post)transcriptional or (post)translational regulation of *Cox6a2* may also be involved in these pathways.

In conclusion, our data show that COX6A2 an important and previously unrecognized role in thermogenesis and whole-body energy metabolism. Therefore, we believe that COX6A2 may be a potential new target for therapy against obesity and/or insulin resistance. However, full body *Cox6a2* ^−/−^ mice display mild cardiac dysfunction and are very sensitive to food deprivation. While the latter may have been the evolutionary driving force to express the protein in wild-type animals, it would compromise a therapy aimed at complete inhibition of COX6A2 activity. Future translational research should therefore aim at partial inhibition of the protein specifically targeting skeletal muscles.

## Experimental Procedures

### Animals and diet

Mice deficient in the *Cox6a2* gene were generated as previously described [30]. Age- and sex-matched C57BL/6J mice (Harlan Laboratories) were used as controls. Mice were fed a regular rodent diet (R/M-H, Ssniff, Soest, Germany). For high-fat diet studies, mice were given a diet containing 45% fat (D12451, Research Diets Inc., New Brunswick, USA) starting from 6 weeks of age, unless otherwise stated. Body weight was measured weekly. Animals were kept on a 12 hr light/12 hr dark cycle at 20°C according to the guidelines approved by the KU Leuven animal welfare committee and the Institutional Animal Care and Research Advisory Committee of the University of Texas Southwestern Medical Center. The ethics committee specifically approved this study with project number p085/2007. For body temperature measurements, rectal temperature was measured at room temperature (time 0) using a DT-610B thermocouple thermometer (ShenZhen Everbest Machinery Industry CO.,Ltd, Shiyan, China). Mice were subsequently deprived of food, caged individually, and transferred to a cold room (4°C). Rectal temperature was then measured every hour for three hours. For the fasting experiment, mice were deprived of food, and caged individually early into the light cycle (8 am). Body weight was then followed for 36 hours.

### Adipocyte size

The mean adipocyte size of subcutaneous and gonadal WAT was determined by computer-assisted image analysis of adipose tissue sections stained with hematoxylin and eosin as described previously [75].

### Glucose tolerance tests

Following an overnight fast (16 h), mice were injected with glucose (2.5 mg/g BW) in the intraperitoneal cavity. Blood samples were drawn from the tail vein at the indicated time points and glucose concentrations were measured using a Glucocard Memory PC (Arkray, Inc., Kyoto, Japan) glucometer.

### Hyperinsulinemic, euglycemic clamps

Clamps were performed as described earlier [76], with modifications. Briefly, after an overnight fast mice were sedated using a mixture of vetranquil, dormicum and fentanyl and a canula was placed in the tail vein. A basal infusion containing 1-^14^C-glucose was subsequently injected for 1 hour at a speed of 50 µl/h. Blood samples were taken after 50 and 60 min for glucose and insulin measurement and tracer dilution. Next, a 30 µl bolus of 100 mU/ml of insulin (Actrapid, Novo Nordisk) was injected i.v., followed by infusion of an hyperinsulinemic solution containing 1-^14^C-glucose at a speed of 50 µl/h. To maintain blood glucose levels, a variable infusion of 12.5% glucose was started and the rate was adjusted to blood glucose levels measured at 5, 10, 15, 20, 30, 40, 50, 60, 70, 80 and 90 min. At 70, 80 and 90 min after the start of the insulin infusion, blood samples were taken for glucose (Hexokinase method, INstruchemie, Delfzijl, The Netherlands) and insulin (Low range Ultra Sensitive Mouse Insulin ELISA kit, Chrystal Chem, Downer's Grove, IL, USA) measurement.

Whole body glucose disposal (µmol/min·kg) was calculated during the basal period and under steady-state clamp conditions as the rate of tracer infusion (dpm/min) divided by the plasma specific activity of 1-^14^C-glucose (dpm/µmol). The ratio was corrected for body weight. The hyperinsulinemic HGP was calculated as the difference between the tracer-derived rate of glucose appearance and the glucose infusion rate.

### Indirect calorimetry

Utilizing the Comprehensive Lab Animal Monitoring System (CLAMS; Columbus Instruments, Columbus, Ohio), *in vivo* metabolic rates were measured in weight-, age-, and sex-matched WT and *Cox6a2* ^−/−^ mice fed a regular diet. The metabolic studies were undertaken as previously described [77].

### Quantitative RT-PCR

Quantitative RT-PCR was performed as described [78]. Gene expression was normalized to RNA Pol II or beta actin expression and the expression level for each gene in WT mice fed a regular diet was set at 1.0. Primer and probe sequences are summarized in Table S4.

### Assessment of Reactive Oxygen Species (ROS) levels

Utilizing the fluorescent probe dihydroethidium (DHE; Molecular Probes/Invitrogen, Carlsbad, California) *in situ* assessment of ROS levels within the diaphragm and hindlimbs of WT and *Cox6a2* ^−/−^ mice was performed. The diaphragm and hindlimbs of 2 to 4 months old male mice were harvested and cryopreserved. Multiple frozen sections (6 µm in thickness, n = 6 in each group) from each muscle group were cut onto glass slides and then incubated with 2 µM DHE in a light-restricted, humidified chamber at 37°C for 30 min. A control set of slides was incubated with a PBS-based vehicle without DHE under similar conditions. Upon completion of the incubation period, vectashield (Vector Laboratories; Burlingame, California) and coverslips were placed over the tissue sections. Assessment of ROS levels was performed utilizing a photomicroscope equipped with fluorescence (Leica DM-2000; Wetzlar, Germany). Quantification of the ROS levels on a tissue section was undertaken by measuring the number of DHE positive nuclei per unit area.

### Metachromatic fiber typing of skeletal myofibers

The gastrocnemius muscles from WT and *Cox6a2* ^−/−^ mice were harvested and cryoembedded in gum tragacanth without fixation and stored at −80°C. Subsequently, fiber typing was performed on cryosections (8 µm thickness) utilizing a metachromatic dye-ATPase assay as previously described [79].

### Myofiber cross-sectional area

Measurements of cross-sectional areas of skeletal muscle fibers were made on images of transverse cryosectioned muscle stained with metachromatic ATPase, and were quantified using NIH software ImageJ 1.37V. The software was used to draw boundaries and calculate areas for any individual cell within a snapshot. The appropriate square micron scale was set up prior to measuring the area. From each snapshot, multiple cells were measured for each of the three fiber types, and multiple snapshots were used per mouse.

### Statistical analysis

All data are shown as means+SEM. Differences between experimental groups were determined by the unpaired (paired when appropriate), two-tailed Student's *t*-test. Whenever the criteria for applying the *t*-test were not met, we used the Mann-Whitney rank-sum test. A *p*-value <0.05 was considered to indicate statistical significance.

Other methods used in this manuscript are available as supporting information (M&M S1).

## Supporting Information

Figure S1 **Total body composition, food intake and thyroid and pituitary hormones of WT** ***versus Cox6a2*** **^−/−^ mice fed a regular diet.** (A–B) % lean mass (A), and % fat mass (B) were assessed by NMR, (C) Food and water consumption measured in 12–16 weeks old mice (n = 4–5). Measurements were performed over a 3-day period, (D) Thyrotropin (TSH) bioactivity (left panel) was measured by a standard bioassay (n = 5). Thyroxine (T_4_) levels (right panel) were assayed by RIA (n = 5). *p<0.05. In all panels, data represent mean+SEM.
(TIF)

Figure S2 **Cox6a2 is not expressed in thermogenic adipose tissues.** (A–B) Left panels: Microarray hybridization signals from public data in BAT (A: GSE7623) and a comparison between mesenteric and SC WAT (B: E-MEXP-1636). Right panels: Correlation between Cox6a2 signals and signals for muscle markers and UCP1 in BAT (A) and SC WAT (B).
(TIF)

Figure S3 **Accelerated body weight loss of fasted** ***Cox6a2*** **^−/−^ mice.** (A–B) Male (A) (n = 10) and female (B) (n = 3–7) mice were deprived of food at 7 am. Throughout the experiment, they had unlimited access to water. Body weight was measured for 36 h. Percentage body weight loss per hour of fasting (slope) is shown on the right. Note that two out of ten male *Cox6a2* ^−/−^ mice were eliminated from the experiment after 24 h, (C) Cox6a2 mRNA expression in soleus muscle, gastrocnemius muscle (gastroc) and diaphragm of overnight fasted (16 h) WT mice. Gene expression in fed mice was set at 1.0 for each individual tissue, (D) Cox6a2 protein abundance in soleus muscle, gastrocnemius muscle (gastroc) and diaphragm of overnight fasted (16 h) WT mice. Cox6a2 protein expression in WT mice was set at 1.0 for each individual tissue. *p<0.05, **p<0.01, ***p<0.001. In all panels, data represent mean ± SEM.
(TIF)

Figure S4 **Ucp1 and Ucp2 mRNA expression in WAT and gastrocnemius muscle of mice fed a HFD.** (A–B) Quantitative RT-PCR was performed on cDNA from white adipose tissue (WAT), (C) and gastrocnemius muscle (gastroc), (D) (n = 5). Gene expression in wild-type mice on a regular diet was set at 1.0 for each individual gene (dashed line). Note that Ucp1 mRNA was not detectable in gastrocnemius muscle. **p<0.01. Data represent mean+SEM.
(TIF)

Table S1 **Plasma parameters in fed WT and** ***Cox6a2*** **^−/−^ mice.**
(PDF)

Table S2 **Gene Set Enrichment Analysis.**
(PDF)

Table S3 **Gene expression of antioxidant enzymes in** ***Cox6a2*** **^−/−^** ***vs*** **WT mice.**
(PDF)

Table S4 **Primers and probes used for quantitative PCR.**
(PDF)

M&M S1 **Supportive Experimental procedures.**
(DOCX)
