# Supplementary figures and images for: Mice Deficient in the Respiratory Chain Gene Cox6a2 Are Protected against High-Fat Diet-Induced Obesity and Insulin Resistance
Source: PLoS One. 2013 Feb 27;8(2):e56719. doi: 10.1371/journal.pone.0056719 (PMC3584060; doi:10.1371/journal.pone.0056719)

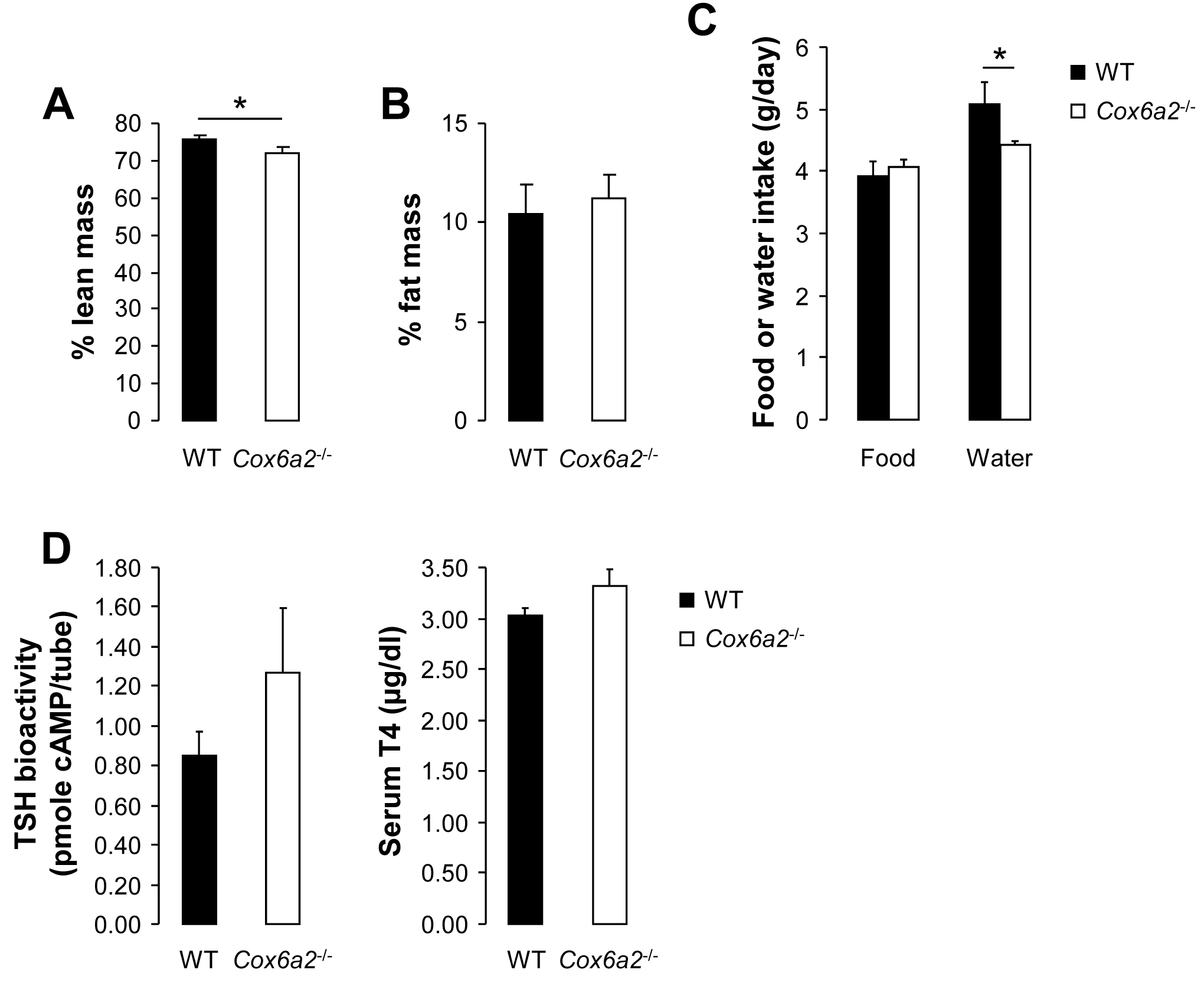

Supplement: Figure S1 — Total body composition, food intake and thyroid and pituitary hormones of WT versus Cox6a2 −/− mice fed a regular diet. (A–B) % lean mass (A), and % fat mass (B) were assessed by NMR, (C) Food and water consumption measured in 12–16 weeks old mice (n = 4–5). Measurements were performed over a 3-day period, (D) Thyrotropin (TSH) bioactivity (left panel) was measured by a standard bioassay (n = 5). Thyroxine (T4) levels (right panel) were assayed by RIA (n = 5). *p<0.05. In all panels, data represent mean+SEM. (TIF) [file pone.0056719.s001.tif]

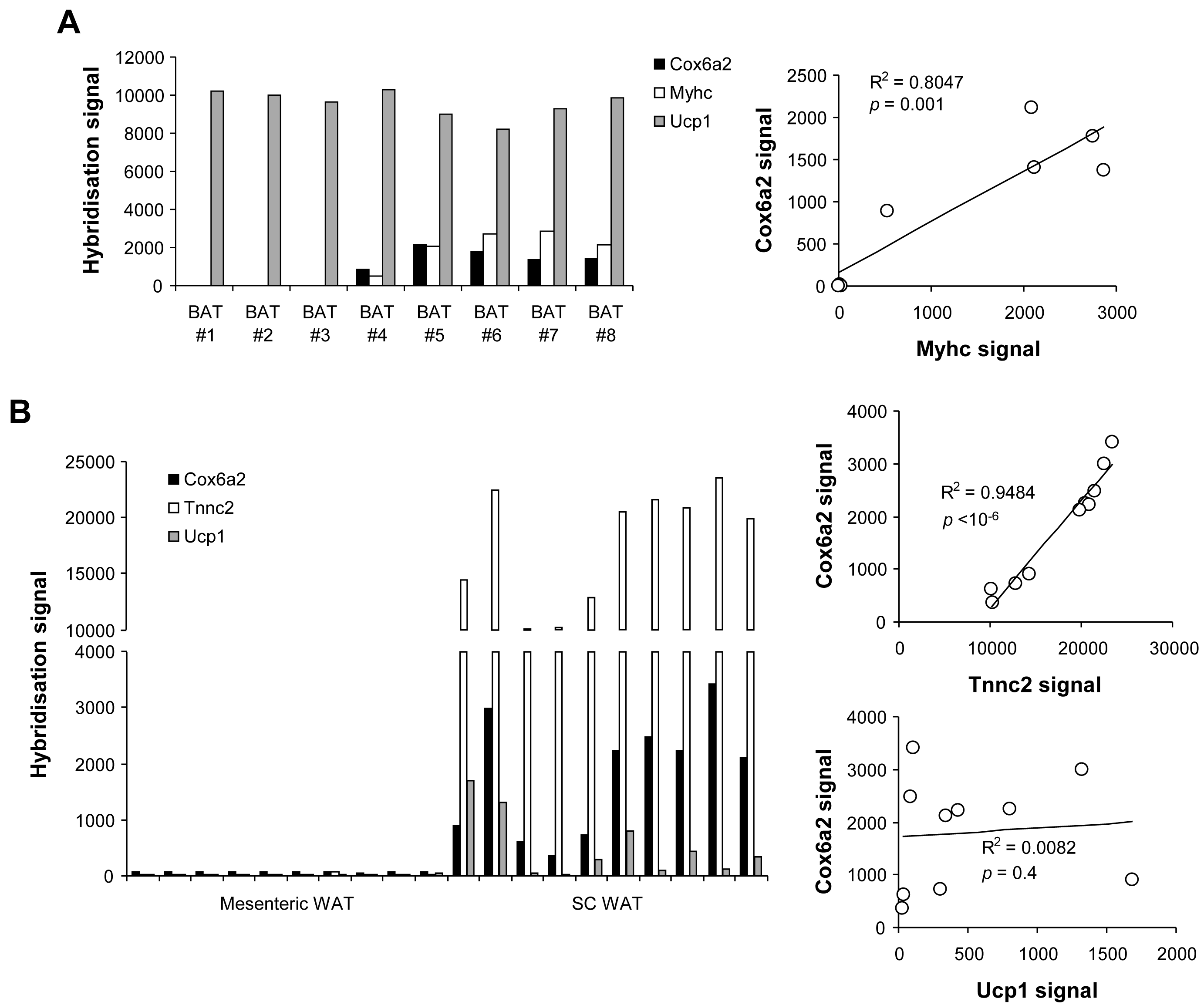

Supplement: Figure S2 — Cox6a2 is not expressed in thermogenic adipose tissues. (A–B) Left panels: Microarray hybridization signals from public data in BAT (A: GSE7623) and a comparison between mesenteric and SC WAT (B: E-MEXP-1636). Right panels: Correlation between Cox6a2 signals and signals for muscle markers and UCP1 in BAT (A) and SC WAT (B). (TIF) [file pone.0056719.s002.tif]

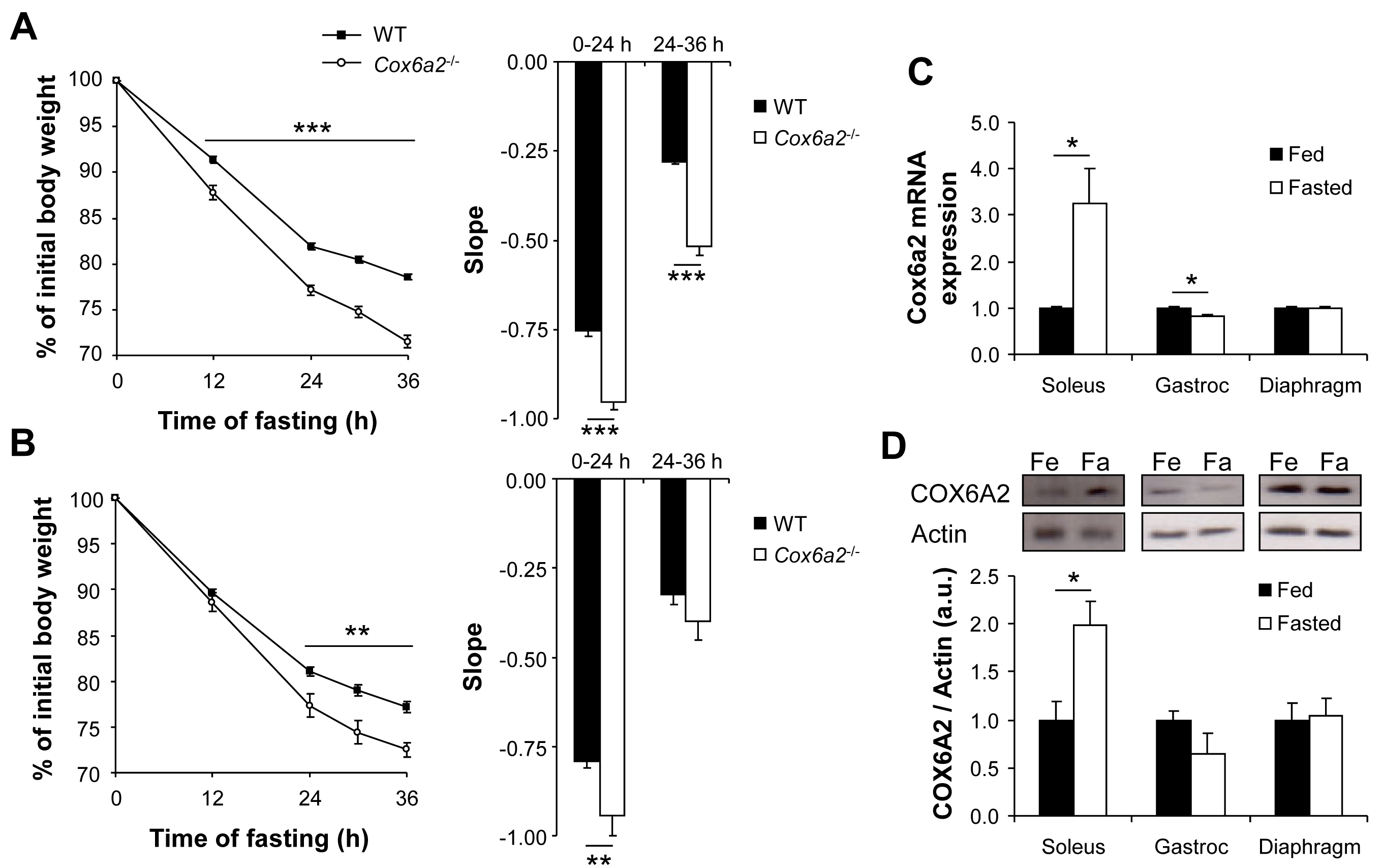

Supplement: Figure S3 — Accelerated body weight loss of fasted Cox6a2 −/− mice. (A–B) Male (A) (n = 10) and female (B) (n = 3–7) mice were deprived of food at 7 am. Throughout the experiment, they had unlimited access to water. Body weight was measured for 36 h. Percentage body weight loss per hour of fasting (slope) is shown on the right. Note that two out of ten male Cox6a2 −/− mice were eliminated from the experiment after 24 h, (C) Cox6a2 mRNA expression in soleus muscle, gastrocnemius muscle (gastroc) and diaphragm of overnight fasted (16 h) WT mice. Gene expression in fed mice was set at 1.0 for each individual tissue, (D) Cox6a2 protein abundance in soleus muscle, gastrocnemius muscle (gastroc) and diaphragm of overnight fasted (16 h) WT mice. Cox6a2 protein expression in WT mice was set at 1.0 for each individual tissue. *p<0.05, **p<0.01, ***p<0.001. In all panels, data represent mean ± SEM. (TIF) [file pone.0056719.s003.tif]

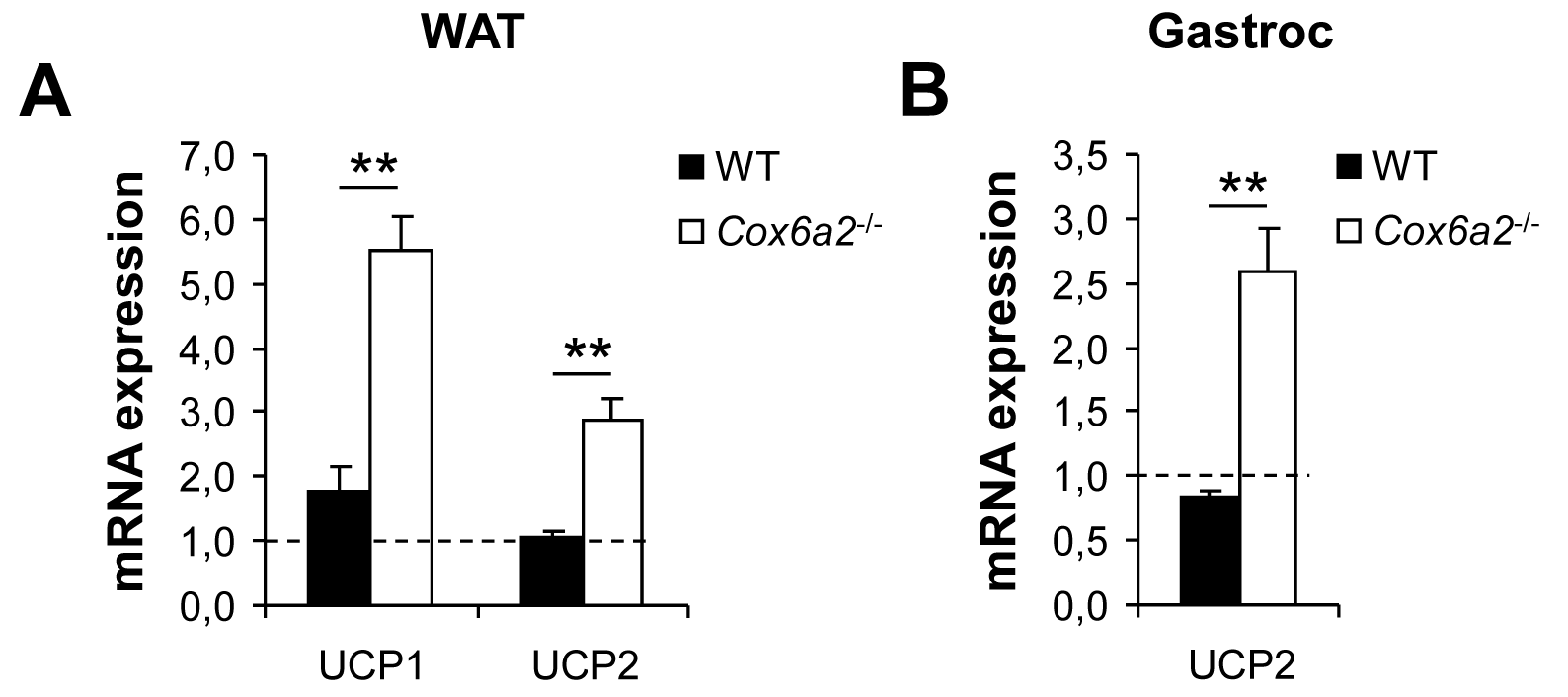

Supplement: Figure S4 — Ucp1 and Ucp2 mRNA expression in WAT and gastrocnemius muscle of mice fed a HFD. (A–B) Quantitative RT-PCR was performed on cDNA from white adipose tissue (WAT), (C) and gastrocnemius muscle (gastroc), (D) (n = 5). Gene expression in wild-type mice on a regular diet was set at 1.0 for each individual gene (dashed line). Note that Ucp1 mRNA was not detectable in gastrocnemius muscle. **p<0.01. Data represent mean+SEM. (TIF) [file pone.0056719.s004.tif]
