# Supplementary material for: Mice Deficient in the Respiratory Chain Gene Cox6a2 Are Protected against High-Fat Diet-Induced Obesity and Insulin Resistance
Source: PLoS One. 2013 Feb 27;8(2):e56719. doi: 10.1371/journal.pone.0056719 (PMC3584060; doi:10.1371/journal.pone.0056719)
Supplement: Table S1 — Plasma parameters in fed WT and Cox6a2 −/− mice. (PDF) [file pone.0056719.s005.pdf]

Table S1: Plasma parameters in fed WT and *Cox6a2*$^{-/-}$ mice

	Regular diet		HFD	
	WT	*Cox6a2*$^{-/-}$	WT	*Cox6a2*$^{-/-}$
Age (weeks)	13	13	18	18
Body weight (g)	21.3 ± 0.6	25.9 ± 1.4[b]	27.5 ± 0.9	39.9 ± 1.9[c]
Glucose (mg/dl)	132.4 ± 4.6	118.8 ± 7.7	133.6 ± 10.8	130.0 ± 9.2
Insulin (ng/ml)	0.9 ± 0.1	0.6 ± 0.1	3.8 ± 1.3[d]	0.7 ± 0.1[a]
Leptin (ng/ml)	ND	ND	43.8 ± 5.5	15.9 ± 2.9[b]
Cholesterol (mg/dl)	ND	ND	163.5 ± 20.7	109.8 ± 4.0[a]
HDL-cholesterol (mg/dl)	ND	ND	140.3 ± 17.6	92.4 ± 3.2[a]
Triglycerides (mg/dl)	ND	ND	72.0 ± 3.7	46.2 ± 2.0[c]
ALT (U/l)	ND	ND	55.5 ± 18.6	33.6 ± 1.1
AST (U/l)	ND	ND	80.3 ± 17.4	44.4 ± 2.4

Data are means ± SEM; n = 5 mice in each group.

ND: Not determined.

[a] $p < 0.05$.

[b] $p < 0.01$.

[c] $p < 0.001$.

[d] $p < 0.05$ versus wild-type mice on regular diet
