# Supplementary material for: Mice Deficient in the Respiratory Chain Gene Cox6a2 Are Protected against High-Fat Diet-Induced Obesity and Insulin Resistance
Source: PLoS One. 2013 Feb 27;8(2):e56719. doi: 10.1371/journal.pone.0056719 (PMC3584060; doi:10.1371/journal.pone.0056719)
Supplement: Table S2 — Gene Set Enrichment Analysis. (PDF) [file pone.0056719.s006.pdf]

Table S2. Gene Set Enrichment Analysis

MSigDB Gene sets enriched in *Cox6a2*$^{-/-}$ diaphragm	Set size	ES	NES	FDR
MITOCHONDRION	318	0.541813	2.408053	0
MITOCHONDRIAL_INNER_MEMBRANE	64	0.646979	2.311377	0
MITOCHONDRIAL_MEMBRANE	83	0.608108	2.277644	0
ORGANELLE_INNER_MEMBRANE	72	0.612554	2.251388	0
OXIDOREDUCTASE_ACTIVITY	253	0.512047	2.215519	4.92E-04
CELLULAR_RESPIRATION	19	0.817346	2.20927	8.37E-04
OXIDOREDUCTASE_ACTIVITY__ACTING_ON_THE_CH_CH_GROUP_OF_DONORS	21	0.770929	2.188349	7.17E-04
MITOCHONDRIAL_PART	137	0.539008	2.180786	6.28E-04
AEROBIC_RESPIRATION	15	0.837027	2.157144	5.58E-04
MITOCHONDRIAL_ENVELOPE	94	0.568591	2.15611	5.02E-04
MITOCHONDRIAL_MEMBRANE_PART	50	0.639882	2.142122	5.69E-04
MITOCHONDRIAL_RESPIRATORY_CHAIN	23	0.733358	2.138296	7.25E-04
COFACTOR_METABOLIC_PROCESS	51	0.610434	2.081699	0.001808
SMALL_GTPASE_REGULATOR_ACTIVITY	66	0.580784	2.069197	0.00212
ELECTRON_CARRIER_ACTIVITY	70	0.565084	2.034657	0.004112
RAS_GUANYL_NUCLEOTIDE_EXCHANGE_FACTOR_ACTIVITY	18	0.734683	2.022251	0.004234
ACTIN_BINDING	66	0.553977	1.979963	0.007334
ENZYME_ACTIVATOR_ACTIVITY	120	0.491528	1.978336	0.0072
TRANSMEMBRANE_RECEPTOR_PROTEIN_PHOSPHATASE_ACTIVITY	19	0.716148	1.97641	0.007015
LIPID_CATABOLIC_PROCESS	35	0.628509	1.958674	0.008562

MSigDB gene sets enriched in *Cox6a2*$^{-/-}$ gastrocnemius muscle	Set size	ES	NES	FDR
MITOCHONDRION	318	0.609892	2.606538	0
MITOCHONDRIAL_PART	137	0.655147	2.594295	0
MITOCHONDRIAL_MEMBRANE	83	0.659844	2.442682	0
MITOCHONDRIAL_ENVELOPE	94	0.656298	2.419941	2.28E-04
MITOCHONDRIAL_INNER_MEMBRANE	64	0.692267	2.411105	1.82E-04
MITOCHONDRIAL_MEMBRANE_PART	50	0.71233	2.407003	1.52E-04
ORGANELLE_INNER_MEMBRANE	72	0.668519	2.381843	2.63E-04
ORGANELLE_ENVELOPE	164	0.583077	2.354623	3.41E-04
ENVELOPE	164	0.583077	2.345933	3.03E-04
GOLGI_VESICLE_TRANSPORT	47	0.678286	2.256097	9.91E-04
ORGANELLE_MEMBRANE	287	0.528384	2.248906	0.001147
MITOCHONDRIAL_MATRIX	44	0.672492	2.203385	0.001795
MITOCHONDRIAL_LUMEN	44	0.672492	2.183654	0.00228
MITOCHONDRIAL_RESPIRATORY_CHAIN	23	0.762388	2.179369	0.002245
ENDOPLASMIC_RETICULUM_MEMBRANE	82	0.590786	2.158575	0.002634
NUCLEAR_ENVELOPE_ENDOPLASMIC_RETICULUM_NETWORK	90	0.571843	2.115288	0.006316
PROTEIN_FOLDING	54	0.616916	2.103335	0.007159
ENDOPLASMIC_RETICULUM_PART	91	0.549838	2.042313	0.01335
OXIDOREDUCTASE_ACTIVITY	253	0.485599	2.039597	0.013068
MITOCHONDRION_ORGANIZATION_AND_BIOGENESIS	44	0.613294	2.038418	0.012414

http://www.broad.mit.edu/gsea/doc/GSEAUserGuideFrame.html?_Interpreting_GSEA_Results
ES: Enrichment Score
NES: Normalized Enrichment Score
FDR: False Discovery Rate
