# Supplementary material for: Mice Deficient in the Respiratory Chain Gene Cox6a2 Are Protected against High-Fat Diet-Induced Obesity and Insulin Resistance
Source: PLoS One. 2013 Feb 27;8(2):e56719. doi: 10.1371/journal.pone.0056719 (PMC3584060; doi:10.1371/journal.pone.0056719)
Supplement: Table S3 — Gene expression of antioxidant enzymes in Cox6a2 −/− vs WT mice. (PDF) [file pone.0056719.s007.pdf]

Table S3: Gene expression of antioxidant enzymes in *Cox6a2*$^{-/-}$ *vs* WT mice

	Diaphragm		Gastrocnemius	
	FC*	*p*-value	FC*	*p*-value
Sod1 (superoxide dismutase 1)	1,05	NS	0.97	NS
Sod2 (superoxide dismutase 2)	1,46	0.01	1.07	NS
Gpx1 (glutathione peroxidase 1)	1,72	0.006	1.03	NS
Gpx3 (glutathione peroxidase 2)	1,07	NS	1.04	NS
Gpx4 (glutathione peroxidase 3)	1.30	0.04	1.23	0.007
Cat (catalase)	1,53	0.004	0.79	0.03
Txn1 (thioredoxin 1)	1,36	0.004	0.96	NS
Txn2 (thioredoxin 2, mitochondrial)	1,14	0.01	1.17	0.0008
Prdx1 (peroxiredoxin 1)	1,02	NS	1.16	NS
Prdx2 (peroxiredoxin 2)	1,95	0.0009	1.08	NS
Prdx3 (peroxiredoxin 3)	1.40	0.003	1.18	0.02
Prdx4 (peroxiredoxin 4)	2,03	0.03	1.23	0.04
Prdx5 (peroxiredoxin 5)	1,32	0.008	1.06	NS
Prdx6 (peroxiredoxin 6)	1,72	0.004	1.11	NS

* Fold change expression in *Cox6a2*$^{-/-}$ over WT mice
