# Supplementary material for: Mice Deficient in the Respiratory Chain Gene Cox6a2 Are Protected against High-Fat Diet-Induced Obesity and Insulin Resistance
Source: PLoS One. 2013 Feb 27;8(2):e56719. doi: 10.1371/journal.pone.0056719 (PMC3584060; doi:10.1371/journal.pone.0056719)
Supplement: Table S4 — Primers and probes used for quantitative PCR. (PDF) [file pone.0056719.s008.pdf]

Table S4. Primers and probes used for quantitative PCR

RNA Pol II	FW	GCACCACGTCCAATGATATTGTG
	Rev	GGAGATGACATGGTACAGTTCTCG
	Probe	(FAM)CTTCCGCACAGCCTCAATGCCCAGT(TAMRA)
Beta actin	FW	AGCCATGTACGTAGCCATCCA
	Rev	TCTCCGGAGTCCATCACAATG
	Probe	(FAM)TGTCCCTGTATGCCTCTGGTCGTAC(TAMRA)
Cox6a2	FW	CGAGCGCCCAGAGTTCATC
	Rev	GATTGTGAAAAGCGTGTGGTTG
	Probe	(FAM)CACCTCCGCATCCGAACCAAGCCCTTC(TAMRA)
Ucp1	FW	CTGCCAAAGTCCGCCTTC
	Rev	GGTCCCTAGGACACCTTTATACC
	Probe	(FAM)ACTGGAAGCCTGGCCTTCACCTTGG(TAMRA)
Ucp2	FW	CCTGAAAGCCAACCTCATGAC
	Rev	GATGACGGTGGTGCAGAAG
	Probe	(FAM)CCTCCCTTGCCACTTCACTTCTGCCTTC(TAMRA)
Pgc-1α	FW	CGAACCTTAAGTGTGGAACTCTC
	Rev	GGTTATCTTGGTTGGCTTTATGAGG
	Probe	(FAM)CTGCAGGCCTAACTCCTCCCACAACTC(TAMRA)
