# Supplementary material for: Mice Deficient in the Respiratory Chain Gene Cox6a2 Are Protected against High-Fat Diet-Induced Obesity and Insulin Resistance
Source: PLoS One. 2013 Feb 27;8(2):e56719. doi: 10.1371/journal.pone.0056719 (PMC3584060; doi:10.1371/journal.pone.0056719)
Supplement: M&M S1 — Supportive Experimental procedures. (DOCX) [file pone.0056719.s009.docx]

**M&M S1: Supportive Experimental Procedures**

### Western blotting

Tissues were isolated from mice, immediately snap-frozen in liquid nitrogen and stored at -80°C until further manipulation. Tissues were homogenized using a glass pestle in icecold lysis buffer (1% NP40, 50 mM Tris pH 8.0, 150 mM NaCl, 0.1 mg/ml PMSF, 150 mM NaF, 0.5 mM Na-orthovanadate, 50 mM B-glycerolphosphate, protease inhibitors (Roche, Mannheim, Germany), and phosphatase inhibitors (Sigma, Steinheim, Germany). Debris was removed by centrifugation for 15 min at 14,000 rpm and 4°C. Protein concentrations were measured using the Dc Protein Assay (BioRAD). Thirty to 40 μg of protein for each sample were electrophoretically separated on a 4-12% Bis-Tris NuPage gel (Invitrogen) using MES buffer (Invitrogen). Proteins were transferred to a PVDF membrane and non-specific sites were blocked in 5% non-fat dry milk in TBS-Tween 0.05% (TBST). Blots were next incubated overnight at 4°C with primary antibody (#MS-411 (Cox6a2) and #MS-412 (Cox6a1) (MitoSciences); sc-74461 (AMPK) (Santa Cruz); #2531 (p-AMPK), #9272 (Akt) and #9275 (p-Akt) (Cell Signaling)). After washing in TBST, blots were incubated with horseradish peroxidase-conjugated secondary antibody for 1 h at room temperature. Densitometry of the bands was performed using ImageJ.

### Cytochrome *c* oxidase activity measurements

The Cox enzymatic activity was determined in isolated mitochondrial preparations from various tissues, using the Cytochrome *c* oxidase assay kit (Sigma CYTOCOX1). This colorimetric assay is based upon the observation of the decrease in absorbance at 550 nm of ferrocytochrome *c* caused by its oxidation to ferricytochrome *c* by Cox.

Tissues were dissected, diced and suspended in isolation medium A (0.22 M mannitol, 0.07 M sucrose, 0.02 M HEPES, 2 mM Tris–HCl, pH 7.2, 1 mM EDTA) and homogenized using a Dounce homogenizer. The suspension was centrifuged at 3000 × *g* for 1.5 min and the pellet was washed in solution A and resuspended in solution B (0.22 M mannitol, 0.07 M sucrose, 0.01 M Tris–HCl, pH 7.2, 1 mM EDTA) and finally centrifuged at 17,500 × *g* for 2.5 min. The pellet was suspended in solution B and protein concentration was estimated. Cox activity was determined by following the manufacturer’s protocol. Briefly, the decrease in absorption at 550 nm of various mitochondrial preparations was followed using a kinetic program with 10 s intervals for a total of six readings. The reaction was started by adding 50 µl of ferrocytochrome *c* to the mitochondrial preparations from different tissues and the decrease in the absorbance was followed. The activity of Cox was calculated and represented per microgram of mitochondrial protein.

### Oxygen consumption from isolated diaphragm

Oxygen consumption rates (OCR) were measured in 96-well plates with an oxygen sensing patch probe (Ocean Optics) contained at the well bottom. Diaphragms, isolated from mice and punched into equal pieces (Ø 3 mm and 1.4 mg average tissue weight), were transferred to the wells where they completely covered the patch probes. Wells were filled with 100 µl medium (DMEM + 2% FBS + 4 mM L-glutamax + 25 mM HEPES + 10 mM EGTA) and maintained at 37°C in standard air with 5% CO_2_. Measurement of oxygen tension was started after a static equilibration period of 15 min. This initial period resulted in stable oxygen tension gradient formation so the rate of oxygen diffusion would equal the rate of oxygen consumption. OCR were computed from the steady-state solution to Fick’s law ^1^, $OCR=\frac{DS\Delta c}{h}$ where *D* is the oxygen diffusion factor in the medium (3 x 10^-5^ cm^2^/s), S is the surface area of the medium exposed to the atmosphere (0.33 cm^2^), *Δc* is the difference in oxygen tension between the air/medium interface and the medium/tissue interface, and h is the diffusion path length (0.3 cm). Saturated dissolved oxygen concentrations were taken as 192 µM. Oxygen probes were calibrated at the onset of each experiment at 37°C, according to the manufacturer’s instructions. Diaphragms were incubated with NaN_3_ to demonstrate the sensitivity of the method. No significant influence of the experimental measurement period on tissue cell viability could be detected.

### Plasma and serum metabolites

Blood was collected by heart puncture and immediately centrifuged for 10 min at 6000 × g and 4°C. Plasma insulin (Ultrasensitive Mouse Insulin ELISA, Crystal Chemistry, Downers Grove, IL, USA) and leptin (R&D Systems Europe, Lille, France) levels were assessed using commercially available kits. Triglyceride, total, HDL, and LDL cholesterol levels were assessed using routine clinical assays. Serum TSH bioactivity was measured by a standard bioassay using Chinese hamster ovary cells stably transfected with human TSH receptor cDNA ^2^. T_4_ serum concentrations were measured by RIA.

### Insulin stimulation of fasted mice

Mice on a regular diet were fasted overnight (18 h). In the morning, mice were anesthesized using 10% pentobarbital and the soleus muscle from one limb (non-insulin-stimulated control) was dissected and snap-frozen in liquid nitrogen. Then, 0.75 mU/g of human recombinant insulin (Humuline Regular, Lilly) were injected intraperitoneally and after 10 min the soleus muscle was removed from the other limb and snap-frozen. Tissues were stored at -80°C until further manipulation.

### Body composition measurements

Utilizing a state of the art QNMR system, the EchoMRI-100 (ECHO Medical Systems, Houston, Texas), *in vivo* assessment of a variety of whole body composition parameters was performed. These parameters included total body fat mass, lean mass, free body fluids, and total body water. Whole body composition measurements were performed on 2-4 month old male WT and *Cox6a2*^-/-^ mice.

### Microarray analysis

Total RNA (100 ng) was used to analyze the mRNA expression via mouse Gene 1.0 ST arrays according to manufacturer’s manual 701880Rev4 (Affymetrix, Santa Clara, CA). Briefly, in the first cycle, double stranded cDNA was prepared with random hexamers tagged with a T7 promoter sequence followed by the generation of cRNA using the GeneChip WT Synthesis and Amplification kit (Affymetrix). cRNA concentration after cleanup was measured with the NanoDrop ND-1000 spectrophotometer (NanoDrop Technologies, DW). In the second cycle, sense oriented single-stranded DNA containing dUTP is generated and the concentration is, after cleanup, measured using the nanodrop. The cRNA is hydrolyzed and the single stranded DNA is fragmented using uracil DNA glycosylase (UDG) and apurinic/apyrimidinic endonuclease 1 (APE1) (GeneChip WT terminal Labeling kit, Affymetrix). The quality of fragmentation (fragments should be between 40 and 70 nucleotides) is checked on the bioanalyzer (Agilent, Waldbronn, Germany). The fragmented DNA is labeled by terminal deoxynucleotidyl transferase (TDT) with the Affymetrix DNA Labeling reagent that is covalently linked to biotin (GeneChip WT terminal Labeling kit, Affymetrix). Labeled DNA was hybridized to the array during 16h at 45°C. The arrays were washed and stained in a fluidics station using the GeneChip hybridization, Wash and Stain kit (Affymetrix) and scanned using the Affymetrix 3000 GeneScanner.

All image files were generated using the Affymetrix GeneChip command console (AGCC). The raw data were analyzed with RMA sketch using the standard settings for Gene 1.0 ST arrays of Expression Console in the AGCC software (Affymetrix). All data files have been deposited in the NCBI Gene Expression Omnibus (GEO, <http://www.ncbi.nlm.nih.gov/geo/>) under accession number GSE41353.

For analysis of the microarray data we used the Gene Set Enrichment Analysis method ^3,4^. Genes were first ranked according to their signal-to-noise ratio. The GSEA algorithm then calculates a running enrichment score (ES) along the ranked gene list, which reflects the degree to which genes in the gene set are overrepresented at the extremes (top or bottom) of the gene list. Gene sets were downloaded from the MSigDB database (<http://www.broad.mit.edu/gsea/msigdb/index.jsp>).

### Transmission electron microscopy

Mice (two from each group) were sacrificed by cervical dislocation and the diaphragm was carefully dissected, washed in PBS and fixed for 1.5 hrs in 2.5% glutaraldehyde in PBS at 25°C. Samples were washed twice with PBS supplemented with 10 mM Hepes at pH 7.4 and subsequently postfixed with osmium tetroxide, stained with uranyl acetate, dehydrated in ethanol and embedded in Epon. Thin sections were imaged on a Tecnai-20 electron microscope (Philips-FEI). Analysis and measurements of electron micrographs (5800 x magnification) were performed using the MetaMorph software. Per mouse, at least 680 individual mitochondria from at least 10 representative EM fields were analyzed.

### Grip strength

Grip strength was evaluated by means of a grid connected to an isometric force transducer (Digital Force Gauge (DFS2), Chatillon). Mice were lifted by their tail and were made to hold the metal grid with all limbs and were pulled backwards until they could not longer hold the grip. Maximal force (g per g body weight) was registered in five consecutive attempts and the results reflected the average of the best three trials.

### *Ex vivo* muscle strength

For measurements of *ex vivo* muscle strength, methods were used as described earlier ^5^ with several modifications. Mice were anesthetized by a percutaneous injection (50 mg/kg) of nembutal. After dissection of m. soleus and m. extensor digitorum longus (EDL) of both hindlimbs, muscles were mounted vertically on a force transducer (HSE, March-Hugstetten, Germany) in organ baths containing a Krebs-Henseleit solution (118 mM NaCl, 25 mM NaHCO_3_, 5 mM KCl, 1 mM MgSO_4_, 1 mM KH_2_PO_4_, 2.5 mM CaCl_2_, and 5 mM glucose), which was continuously gassed with a mixture of 95% O_2_ and 5% CO_2_ and maintained at 25°C. After mounting, the muscles were allowed to recover from the dissection procedure during a 15 min stabilization period. Muscles were subsequently *supra* maximally stimulated (120%) using field stimulation with platinum electrodes and capacitor discharges of alternating polarity. Stimulations were evoked by an Universal Isolated Stimulator Output (HSE, March-Hugstetten, Germany), which was controlled by a computer program designed by Simulink (Matlab, The Mathworks, Inc., Natick, USA). Optimal force generating length (L_0_) was determined via a micropositioner by stimulating the soleus and EDL tetanically for 1 s at 50 Hz and 0.5 s at 100 Hz, respectively, interspersed with 2 min rest intervals. Muscle contractile properties were then assessed.

### Muscle Isometric Contractile Parameters

Specific muscle force, known as maximal tension (P_peak_) is expressed as the ratio of the measured force (F_0_) and the Cross sectional Area (CSA). CSA is measured by dividing the muscle weight by the product of the muscle length (L_0_) and the muscle density (assumed to be 1.06 mg / mm³).

***Twitch Tension Measurements.*** P_peak_ was studied by stimulating the soleus and EDL three times at 1 Hz, interspersed with 1 min rest intervals. The twitch tension is expressed as the average of three contractions.

***Tetanic Tension Measurements.*** P_peak_ was studied by stimulating the soleus for 700 ms (50 Hz) and EDL for 500 ms (100 Hz) three times interspersed with 2 min rest intervals. Tetanic tension is expressed as the average of three contractions.

***Fatigue Test and Recovery.*** Tetanic force during fatigue was studied using a fatiguing stimulation protocol adapted from Lännergren and Westerblad ^6^. Soleus muscle (50 Hz) was stimulated repetitively for 350 ms in twelve successive 1 min windows with decreasing rest intervals every 2 min (3.8, 3.1, 2.6, 2.1, 1.6, and 1.3 s). To evaluate the muscle fatigue resistance, a 350-ms tetanic contraction was recorded at the end of each 1 min window and after 5 and 10 min of recovery.

### Treadmill experiment

After two 10 min runs on two consecutive days to habituate the mice to the protocol, uphill and downhill running tests were performed on two consecutive days. Endurance tests were performed at an initial velocity of 6 m/min and an increase in velocity of 2 m/min every 5 min. Mice ran at a 10° incline (up- or downhill). The uphill run recruits primarily oxidative muscle fibers, whereas the downhill run mainly recruits glycolytic muscle fibers.

### Muscle ATP content

Skeletal muscles (gastrocnemius, diaphragm, soleus) were dissected from anesthesized mice and immediately frozen in liquid nitrogen. ATP extraction was performed by homogenization in 1.0 ml of icecold 0.4 M perchloric acid. Homogenates were left on ice for 30 min and centrifuged at 18,000 × *g* at 4°C for 10 min. Pellets were saved for protein measurement using the BioRAD Dc protein assay after extraction in cell lysis buffer (1% NP40, 50 mM Tris pH 8.0, 150 mM NaCl, 150 mM NaF, 0.5 mM Na-orthovanadate, 50 mM B-glycerolphosphate, 0.1 mg/ml PMSF, 1% phosphatase inhibitor cocktail II (Sigma), Mini EDTA-free protease inhibitor cocktail (Sigma)) and supernatant was neutralized with 1/10 volume of 4 M K_2_CO_3_. Samples were left on ice for 10 min and subsequently incubated at -80°C for 1-2 h. Mixtures were again centrifuged as above and supernatants were kept at -80°C until ATP measurement using the Cell Titer-Glo luciferase assay (Promega).

1. Guarino, R. D. *et al.* Method for determining oxygen consumption rates of static cultures from microplate measurements of pericellular dissolved oxygen concentration. *Biotechnol. Bioeng.* **86**, 775–787 (2004).

2. Moeller, L. C. *et al.* Hypothyroidism in thyroid transcription factor 1 haploinsufficiency is caused by reduced expression of the thyroid-stimulating hormone receptor. *Mol. Endocrinol* **17**, 2295–2302 (2003).

3. Mootha, V. K. *et al.* PGC-1alpha-responsive genes involved in oxidative phosphorylation are coordinately downregulated in human diabetes. *Nat. Genet.* **34**, 267–273 (2003).

4. Subramanian, A. *et al.* Gene set enrichment analysis: a knowledge-based approach for interpreting genome-wide expression profiles. *Proc. Natl. Acad. Sci. U.S.A.* **102**, 15545–15550 (2005).

5. Eijnde, B. O., Lebacq, J., Ramaekers, M. & Hespel, P. Effect of muscle creatine content manipulation on contractile properties in mouse muscles. *Muscle Nerve* **29**, 428–435 (2004).

6. Lännergren, J. & Westerblad, H. Force decline due to fatigue and intracellular acidification in isolated fibres from mouse skeletal muscle. *J. Physiol. (Lond.)* **434**, 307–322 (1991).
